# Programmable Nanostructure Assembly of a Paclitaxel Derivative Enables Tunable Anticancer Therapy via Hydrogen Bond Engineering

**DOI:** 10.1021/acsnano.5c10267

**Published:** 2025-08-26

**Authors:** Guobing Feng, Hui Tang, Shuyi Xie, Yingying Wang, Tongyu Wu, Xiongru Cai, Yunyi Zhou, Yan Lu, Yuancheng Bai, Mengfan Zhao, Shuai Hu, Yuezhou Zhang, Mohammad-Ali Shahbazi, Hélder A. Santos, Jin Fan, Dongfei Liu

**Affiliations:** † State Key Laboratory of Natural Medicines, School of Pharmacy, 56651 China Pharmaceutical University, Nanjing 210009, China; ‡ Hangzhou Geriatric Hospital, Department of Pharmacy, Affiliated Hangzhou First People’s Hospital Chengbei Campus, School of Medicine, Westlake University, Hangzhou 310022, China; § Department of Orthopaedics, The First Affiliated Hospital of Nanjing Medical University, Nanjing 210029, China; ∥ Department of Biomaterials and Biomedical Technology, The Personalized Medicine Research Institute (PRECISION), 10173 University Medical Center Groningen, University of Groningen, Ant. Deusinglaan 1, Groningen 9713 AV, The Netherlands; ⊥ Frontiers Science Center for Flexible Electronics, Shaanxi Institute of Flexible Electronics, 26487 Northwestern Polytechnical University, Xi’an 710072, China

**Keywords:** hydrogen bonds, assembly morphology, antitumor agents, renal accumulation, self-assembly

## Abstract

Precise control of the morphology of self-assembling drugs is critical for optimizing their pharmacokinetics and therapeutic efficacy. However, adapting a single drug for diverse therapeutic applications by tailoring its structure remains a central challenge. Here, we report a hydrogen-bond-guided strategy to program the morphology of a paclitaxel derivative, PTP, by introducing a phosphate group to promote supramolecular organization. PTP molecules spontaneously formed nanofibers in aqueous environments via directional hydrogen bonding. Through rational coassembly with polyethylene glycol 400 or hyaluronic acid, the nanofibers were, respectively, transformed into spherical nanoparticles (PTP@PEG) or bundled fibers (PTP@HA), enabling tailored pharmacological performance. PTP@PEG enhanced systemic circulation, reduced renal accumulation, and improved antitumor efficacy in a murine 4T1 breast cancer model following intravenous administration. In contrast, PTP@HA exhibited sustained release and potent therapeutic effects in a peritoneal metastasis model of colorectal cancer via intraperitoneal injection. This work demonstrates how tunable hydrogen bonding enables precise programming of drug assembly morphology, offering a versatile platform to expand the therapeutic applications of a single drug across multiple diseases. Tuning the nanostructure of one drug using simple excipients via hydrogen bonds presents a simple and effective approach over designing new carriers, potentially revitalizing drugs previously limited by suboptimal pharmacokinetic or pharmacodynamic profiles.

## Graphical abstract

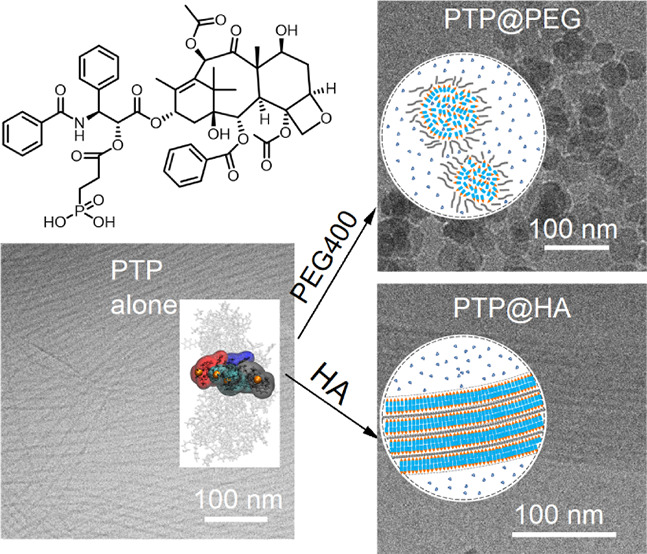

## Introduction

The precise control over material morphology—encompassing size,^1,2^ shape,^3,4^ topology,^5,6^ and hierarchical organization^7–9^ —is fundamental to tailoring their physical, chemical, and biological properties, representing a cornerstone of modern materials science. For instance, in catalysis,^10,11^ tuning nanoparticle morphology can dramatically enhance reaction rates and selectivities by exposing specific active facets. Similarly, in electronics,^12,13^ engineered nanostructures improve charge transport and device efficiency. Consequently, strategies for crafting materials with tailored morphologies, from nanoparticles to hierarchical assemblies, drive innovations across diverse applications, including high-performance batteries,^14,15^ sensors,^16,17^ and pharmaceuticals.^18,19^ This pivotal role of morphology underscores the need for robust, reproducible methods to direct self-assembly and structural organization across multiple length scales.^20–22^

In nanomedicine, the morphology of nanoscale drug delivery systems profoundly impacts on their circulation time, cellular uptake, and tissue penetration, ultimately shaping therapeutic efficacy and side effects.^23–25^ Thus, precise morphology control is essential for optimizing *in vivo* performance and is a key focus of nanobiotechnology strategies.^26–29^ Engineered assemblies, like polymer-based nanocarriers, can adopt diverse shapes to meet varied therapeutic requirements, thereby improving the pharmacological profiles and therapeutic efficacy of loaded cargos.^30,31^ Examples include micellar or nanoparticulate systems that overcome poor water solubility and permeability,^32,33^ surface-functionalized nanorods with controlled biodistribution,^34^ and nanofibers offering sustained drug release via meshed architectures.^35,36^ Despite these advantages, reliably directing nanocarrier morphology remains a major challenge, particularly in drug delivery, where formulation-dependent variability hinders clinical translation.^37^

Designing a single drug to fulfill multiple disease indications poses a daunting challenge, as each pathological context may demand a different morphology for optimal delivery.^38–40^ Certain conditions benefit from elongated nanostructures that facilitate deeper tissue penetration and prolonged retention, whereas others require compact assemblies for efficient cellular uptake.^41,42^ Moreover, even slight variations in formulation parameters (e.g., solvent composition, temperature, or mixing protocol) can drastically shift the resulting shape and size, complicating both reproducibility and large-scale manufacturing.^43^ Among the noncovalent interactions, hydrogen bonding stands out for its directionality and moderate strength, making it highly effective in guiding molecular assembly.^44–46^ By strategically grafting hydrogen-bond donor and acceptor moieties to the drug molecules or excipients, self-assembly pathways can be controlled to yield distinct nanostructures with unique physicochemical properties.^47,48^ Nevertheless, leveraging hydrogen bonding specifically to develop versatile drug delivery platforms remain underexplored.

Recognizing the challenges in realizing the full therapeutic scope of multifaceted compounds, we selected paclitaxel (PTX), a natural product renowned for its complex structure and potent bioactivity,^49,50^ as a model to explore hydrogen-bond-driven morphological control. While traditionally employed for microtubule stabilization and cell division inhibition,^51^ the capacity of PTX to modulate immune responses,^52^ promote axonal regeneration,^53^ and inhibit vascular growth^54^ underscores its multitarget promise, often hindered by delivery obstacles. To unlock this versatility, we engineered an amphiphilic PTX derivative by introducing a hydrophilic phosphate group, enabling robust hydrogen bonding and subsequent nanofiber self-assembly. Deliberate tuning of these interactions allowed precise control over coassembly systems, leading to either disruption or reinforcement of nanofiber formation. This command over supramolecular morphology directly governed pharmacokinetics, tissue distribution, and drug release, consequently impacting pharmacodynamics. This study thereby demonstrates how strategic morphological engineering of a single drug can unlock its therapeutic versatility, enabling tailored efficacy against distinct diseases.

## Results and Discussion

### Hydrogen-Bonding-Driven Self-Assembly of a PTX Derivative with Preserved Bioactivity

To effectively utilize hydrogen bonding for self-assembly and morphology control, we performed a computational screening to identify a diverse set of hydrophilic groups suitable for conjugation to PTX. This screening process, based on assessing hydrogen bonding potential, initially selected five candidate functional groups (R_1_-R_5_). These five candidates were chosen to represent a range of hydrophilic functionalities anticipated to promote robust hydrogen bonding. Subsequently, point charge calculations revealed significant charge differences between the positive and negative charges for functional groups R_3_, R_4_ and R_5_ (Figure 1a), indicating their potential for strong hydrogen bonding interactions.

**Figure 1:**
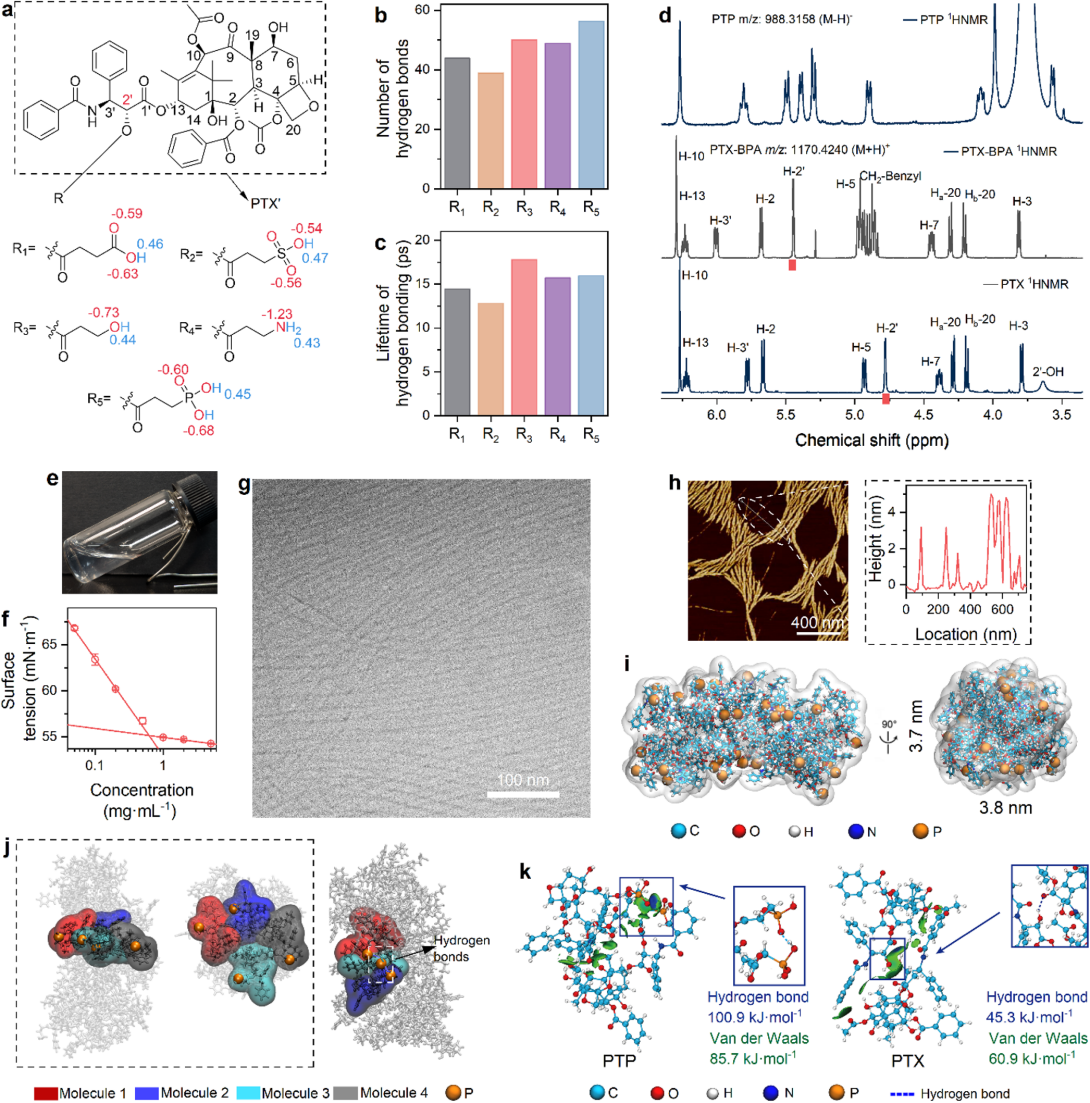
Design, synthesis, and self-assembly of a PTX derivative–PTP. (**a**) The molecular structures of five PTX derivatives for computational screening on hydrogen bonding potential. (**b** and **c**) Number of hydrogen bonds (b) and lifetime of hydrogen bonding (c) in corresponding PTX derivatives. (**d**) ^1^H NMR spectra of PTX, the intermediate PTX-BPA and PTP, highlighting chemical shift changes upon conjugation. (**e**) Photograph of PTP dissolved/dispersed in water at 5.0 mg·mL^–1^. (**f**) Surface tension measurements of PTP dissolved/dispersed in water as a function of concentration, showing a critical aggregation concentration of 0.6 mg·mL^–1^. (**g**) Cryo-TEM image of PTP system in water at 5.0 mg·mL^–1^. (**h**) AFM image (left) and height profile (right) of the PTP nanofibers at 5.0 mg·mL^–1^, confirming their dimensions. (**i**) The molecular assembly structures of 32 PTP molecules self-assembling into a cylindrical nanofiber (front and side views). (**j**) Schematic representation of the representative layered and interlayered structure within the PTP nanofiber, showing the arrangement of hydrophobic PTX’ and hydrophilic PA fragments. (**k**) Interaction energy calculations showing the relative strengths of hydrogen bonding between PA fragments, van der Waals interactions between PTP molecules, and hydrogen bonding between PTX molecules.

To further explore the self-assembly of these conjugates, molecular dynamics simulations were conducted with 50 randomly distributed molecules in water. After a 150 ns simulation, PTX conjugated with R_5_ exhibited the highest number of hydrogen bonds at equilibrium (Figure 1b). The hydrogen bonding lifetimes for PTX conjugated with R_3_, R_4_ and R_5_ were calculated as 17.8 ps, 15.7 and 16.0 ps, respectively (Figure 1c). These lifetime values demonstrated a trend consistent with the point charge calculations. In our analysis, we considered both hydrogen bond density (the number of interactions) and lifetime (their stability). For supramolecular self-assembly, overall cohesive strength is not dictated by a single factor, but rather by the cumulative effect of all interactions. A robust assembly requires a favorable balance between the number of bonds and their longevity. Although the hydrogen bond lifetime for R_5_ is marginally shorter than that of R_3_, the considerably higher number of hydrogen bonds formed by R_5_ at equilibrium is considered a more dominant factor in establishing strong intermolecular interactions and robust self-assembly. Based on these computational results, PTX conjugated with R_5_ was identified as the most promising candidate for subsequent synthesis and experimental validation due to its superior hydrogen bonding capacity and potential for robust self-assembly.

To implement the findings, we conjugated the 2’-position of PTX with the hydrophilic phosphate group, 3-phosphonopropanoic acid (PA), to synthesize the PTX derivative PTP (with the PTX segment in PTP referred to as PTX’). However, direct esterification of PTX and PA produced only a limited yield of PTP (ca. 5.0%), likely due to the hydrophilicity of PA. To overcome this limitation, benzyl groups were introduced to the two hydroxyl groups of PA, forming the more hydrophobic intermediate 3-(bis­(benzyloxy)­phosphoryl)­propanoic acid (BPA).^55–57^ Following the reaction of PTX with BPA, the chemical shift of H-2’ increased by 0.67 ppm (from 4.78 to 5.45 ppm), H-3′ increased by 0.23 ppm (from 5.78 to 6.01 ppm), while H-7 increased by 0.06 ppm (from 4.39 to 4.45 ppm) (Figure 1d).^58^ Detailed assignments for PTP ^1^H NMR (Figure 1d) were omitted due to water peak (δ ∼ 3.5–4.0 ppm) overlap obscuring key proton signals. Subsequent debenzylation afforded PTP with a significantly improved yield of 70.6% (Scheme S1). Structural confirmation of intermediates and the final product was achieved via nuclear magnetic resonance (NMR) and mass spectrometry (MS) analyses (Figures S1–S7). The chemical purity of PTP was confirmed by both ^1^H NMR (Figure S6), which showed no residual benzyl signals, and high-performance liquid chromatography (HPLC) analysis (Figure S8), which indicated a purity above 98%, ensuring synthetic reliability.

Due to the introduction of hydrophilic groups, PTP may exhibit enhanced water solubility and potential amphiphilic properties. As anticipated, when dissolved or dispersed in water at a concentration of 5.0 mg·mL^–1^, the PTP system appeared as a transparent solution (Figure 1e). At concentrations below 1.0 mg·mL^–1^, the water–air surface tension decreased continuously with increasing concentration (Figure 1f). The reduction in water–air interfacial tension confirmed the amphiphilic nature of PTP and indicated its self-assembly behavior. Above 1.0 mg·mL^–1^, the surface tension stabilized at approximately 55.0 mN·m^–1^. The critical aggregation concentration of PTP was determined to be 0.6 mg·mL^–1^, based on the inflection point in the surface tension versus log PTP concentration plot.^59^

Microscopic analyses provided direct evidence for the self-assembly behavior of PTP molecules. The cryogenic transmission electron microscopy (Cryo-TEM) image (Figure 1g) revealed the characteristic morphology of the nanofibers formed by the amphiphilic PTP molecules, displaying a well-organized and aligned arrangement. These nanofibers had diameters of approximately 5 nm and lengths exceeding several hundred nanometers (Figure S9). The atomic force microscopy (AFM) image further confirmed the nanofiber structure and provided topographical information. The height profile extracted from the atomic force microscopy image (right panel of Figure 1h) indicated the formation of nanofibers with diameters of approximately 5 nm, further supporting the formation of consistent, well-defined nanostructures. These results strongly confirm the self-assembly of PTP molecules into nanofibers in aqueous solutions.

The molecular dynamics simulation result revealed a clear visualization of the compact, organized arrangement of 32 PTP molecules, confirming their self-assembly into well-defined cylindrical nanostructures, approximately 4 nm in diameter (Figure 1i). PTP tended to stack at both ends in a layered structure, with all the PA fragments localized on the outer layer. Analysis of a representative four-molecule PTP structure elucidated the organizational principles: hydrophilic PA fragments were oriented outward, while hydrophobic PTX’ fragments formed the central core of the cylindrical fiber (Figure 1j). The cylindrical morphology of PTP nanofibers was primarily stabilized by intermolecular hydrogen bonds between PA groups on adjacent layers. These directional PA–PA interactions aligned with the outward orientation of PA fragments in the stacked structure and were key drivers of fiber elongation along the assembly axis. In parallel, intermolecular hydrogen bonds between PA and PTX’ provided conformational stability by organizing each PTP molecule into an assembly ready geometry (Figure S10). This interaction positioned the hydrophilic PA groups outward and buried the hydrophobic PTX’ segments in the core. By prealigning molecular components, PTP established a structural framework that facilitated efficient intermolecular bonding. Together, PA–PTX’ and PA–PA interactions enabled and sustained the directional growth of well-ordered nanofibers.

Interaction energy calculations further quantified the dominant role of hydrogen bonds in PTP assembly (Figure 1k). Between PTP molecules, hydrogen bonding between PA groups (100.9 kJ·mol^–1^) was found to be stronger than the van der Waals interactions (85.7 kJ·mol^–1^). Critically, when compared to the intrinsic hydrogen bonds between PTX molecules (45.3 kJ·mol^–1^), the strategically grafted PA fragment also significantly enhanced hydrogen bonding strength. These findings indicate that hydrogen bonds, particularly those formed by the PA groups, are the primary stabilizing force in the layered structure of PTP fibers. This stabilization, oriented along the fiber extension direction, drives the formation of the observed cylindrical morphology.

To assess the impact of PA conjugation on the antiproliferation activity of PTX, we evaluated the antiproliferative effects of PTP (in cell culture medium) and PTX (formulated with Cremophor EL and ethanol) in four cancer cell lines. Both compounds exhibited similar dose-dependent antiproliferative activity across all cell lines tested (Figure 2a). The IC_50_ values, representing the concentration required to inhibit cell growth by 50%, were in the nanomolar range for both PTP and PTX (Figure 2b). Specifically, the IC_50_ values for PTP and PTX were 12.5 nM and 13.1 nM in human colon HCT116-Luc cells, 31.2 nM and 37.4 nM in human ovarian SKOV3 cells, 82.4 nM and 25.9 nM in mouse breast 4T1 cells, and 13.2 nM and 11.3 nM in human lung A549 cells, respectively. These results demonstrate that the conjugation of PA to PTX did not compromise its antitumor activity.

**Figure 2:**
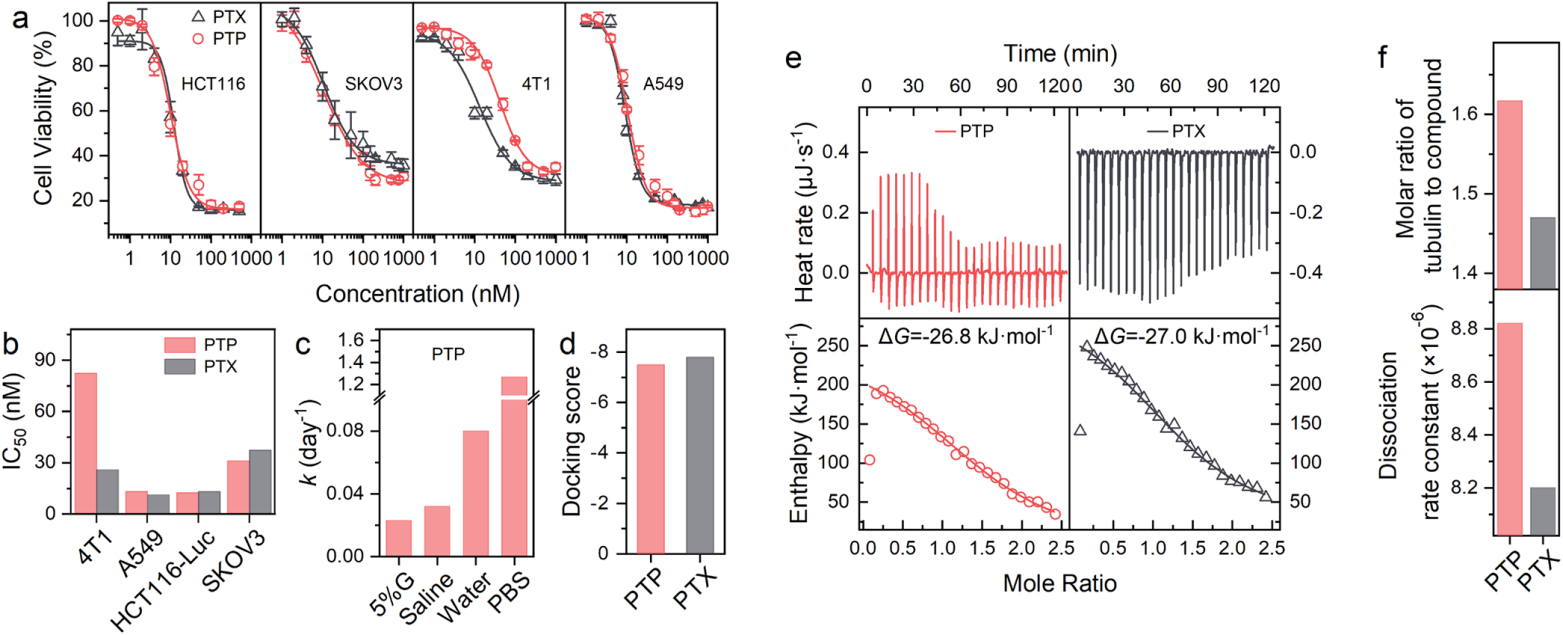
PTP showed antiproliferation effects similar to PTX. (**a** and **b**) Dose–response curves (a) and IC_50_ values (b) for PTP and PTX in four cancer cell lines (HCT116, SKOV3, 4T1 and A549). (**c**) Degradation rate constants of PTP (0.1 mg·mL^–1^) in four aqueous solutions. (**d**) *In silico* docking scores of PTP and PTX with tubulin, predicting their binding affinity. (**e**) ITC data for the interaction of tubulin with PTP and PTX. *Top*: Raw ITC data before background subtraction. *Bottom*: Integrated heat peaks plotted against the molar ratio of tubulin to PTP or PTX. (**f**) Molar ratios and dissociation constants (*K*_d_) for the interaction of tubulin with PTP and PTX, derived from the ITC data.

Given the susceptibility of the ester bond between PA and PTX’ to hydrolysis, we investigated the stability of PTP at 0.1 mg·mL^–1^ in four aqueous solutions. The degradation rate constant (*k*) values were 2.3 × 10^–2^ day^–1^, 3.2 × 10^–2^ day^–1^, 8.0 × 10^–2^ day^–1^, and 1.3 day^–1^ in 5% glucose (5% G), saline, water, and phosphate buffer saline (PBS), respectively (Figures 2c and S11). The degradation product of PTP in PBS was confirmed to be PTX by ^1^H NMR, ^13^C NMR, and MS analyses (Figure S12).^58^ Additionally, a preliminary stability assessment revealed that PTP could be stored at −20 °C for 6 months with minimal degradation, as evidenced by a decrease in purity from 98.5% to 98.1% (Figure S13). The antiproliferation assay was performed at concentrations ranging from 1.0 to 1000.0 nM (0.99 ng·mL^–1^ – 0.99 μg·mL^–1^) in RPMI-1640 or DMEM supplemented with 10% fetal bovine serum, which are several orders of magnitude below the critical aggregation concentration (0.6 mg·mL^–1^). Under these conditions, PTP remains monomeric, undergoes cellular uptake, and is cleaved intracellularly to release active PTX, resulting in growth inhibition comparable to that of free PTX. Therefore, the antiproliferative effect observed in culture media is independent of the degradation behavior of nanofiber assemblies in buffer solutions.

To understand the interaction of PTP with tubulin, the cellular target of PTX, we performed *in silico* docking and isothermal titration calorimetry (ITC) experiments. The docking scores of tubulin with PTP (−7.5) and PTX (−7.8) were similar (Figure 2d), suggesting their comparable binding affinities. However, ITC experiments were challenging due to PTX’s poor water solubility and PTP’s tendency to self-assemble at high concentrations, which limited the achievable drug concentrations. To address this, we adopted an unconventional approach by titrating a tubulin solution (180 μM) into a 5% DMSO (v/v) solution containing 25 μM PTP or PTX. Although this method did not provide a precise characterization, the resulting ITC profiles, lacking the sinusoidal curves, offering valuable insights into the compound-tubulin interaction. The calculated Gibbs free energy change (Δ*G*) values for PTP and PTX were −26.8 kJ·mol^–1^ and −27.0 kJ·mol^–1^, respectively (Figures 2e and S14), indicating favorable interactions with tubulin. Moreover, the molar ratios of tubulin to PTP and PTX were 1.6 and 1.5, respectively, with dissociation constants (*K*_d_) of 8.8 × 10^–6^ and 8.2 × 10^–6^ (Figure 2f). These findings suggest that the introduction of hydrogen bonds through PA conjugation did not compromise PTP’s ability to interact with tubulin and exert its antitumor activity.

The morphology of a drug delivery system significantly influences the behavior of incorporated drugs within the body. To investigate this relationship, we designed an experiment to modulate the morphology of PTP assemblies by strategically employing excipients that interact with their foundational hydrogen bonds. Recognizing that PTP’s fibrous structure is formed through hydrogen bonding between its components, we selected two sets of excipients based on their opposing functionalities: those that disrupt and those that reinforce the assembly. To disrupt the existing PTP structure, we utilized excipients known for their ability to form competing hydrogen bonds, including polyethylene glycol 400 (PEG400), Cremophor EL (CrEL), and Tween 80 (T80).^60^ Conversely, to reinforce the PTP assembly, we selected excipients with established gel-forming properties, namely hyaluronic acid (HA), poloxamer 188 (P188), and chitosan (CS). This deliberate selection of excipients with contrasting effects on hydrogen bonding was designed to demonstrate the impact of these carefully chosen ingredients impact the overall structure of the PTP assemblies and their subsequent behavior.

### Control of Assembly Morphology via Hydrogen Bonding

We initially focused on the three ingredients expected to disrupt PTP assembly structure: PEG400, CrEL, and T80 (Figure 3a). Molecular dynamics simulations were employed to model their interactions with PTP and understand their effects on its structure (Figure S15). Our simulations measured the duration and quantity of hydrogen bonds formed between each ingredient and PTP. PEG400 formed the most hydrogen bonds, and these bonds persisted the longest, indicating a strong interaction with PTP. Specifically, the average lifetime of hydrogen bonding for PEG400 was 24.3 ps (Figure 3b), with an average number of 31.3 bonds during the final 5 ns of the simulation (Figures 3c and S16). In contrast, CrEL and T80 exhibited shorter bonding lifetimes and fewer bonds. The change in solvent-accessible surface area (ΔSASA) from the initial state to the final 5 ns was 10.4% for PEG400, 5.6% for CrEL, and 6.5% for T80 (Figures 3d and S17). While a larger ΔSASA typically indicates more structural alteration, in this context. PEG400s higher value reflects its more extensive interaction with PTP, as evidenced by the greater number and longer duration of hydrogen bonds. Importantly, both CrEL and T80 have been linked to significant side effects in clinical settings,^60,61^ further supporting the choice of PEG400 for controlling the structure of the PTP assemblies. Considering PEG400s strong interaction with PTP, coupled with its established safety profile, it was selected as the most suitable candidate for further investigation.

**Figure 3:**
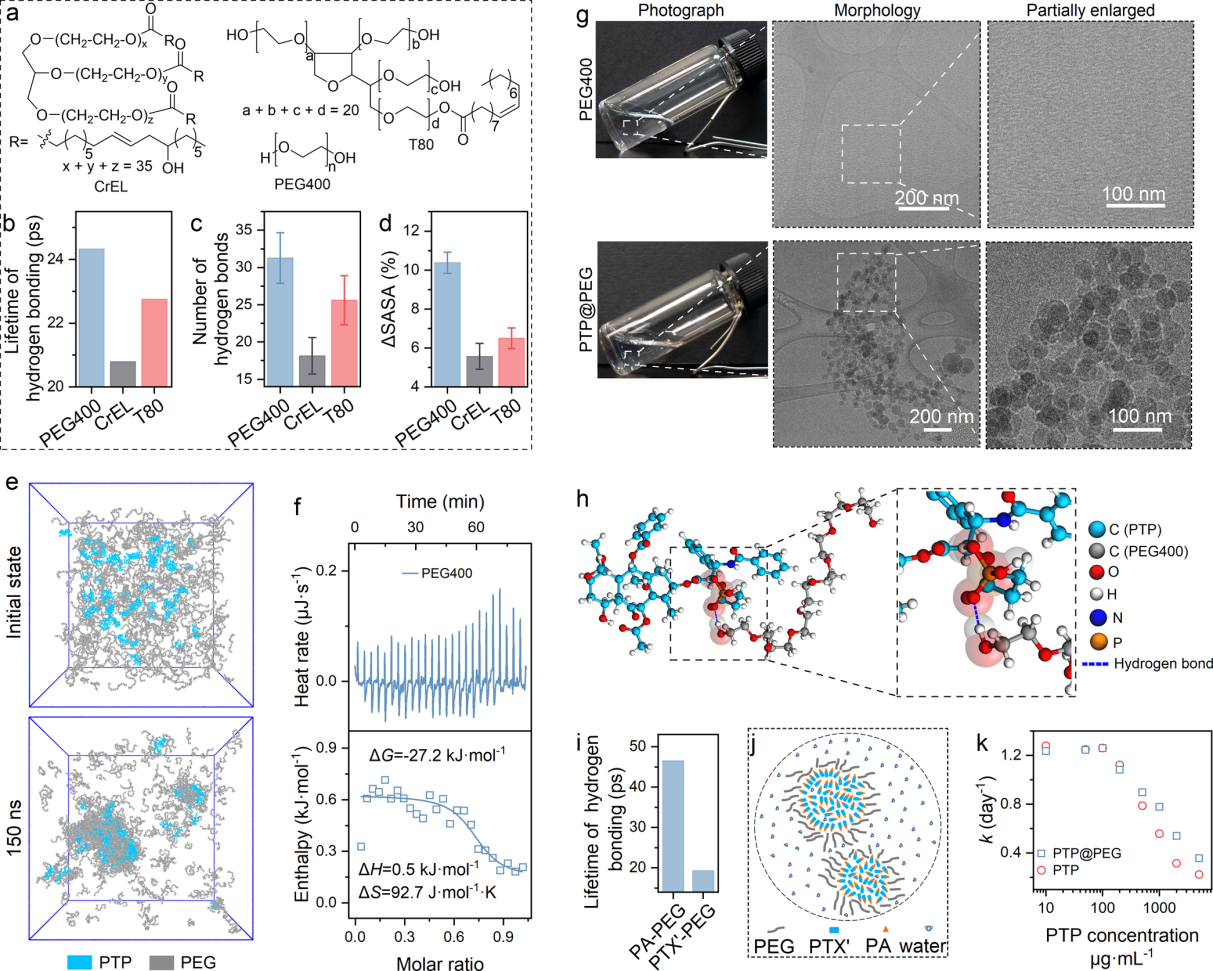
Transformation of PTP morphology into nanoparticles by PEG400. (**a**) The structures of PEG400, CrEL, and T80. (**b** and **c**) Average lifetime of hydrogen bonding (b) and number of hydrogen bonds (c) between PTP and each of the following: PEG400, CrEL, and T80. (**d**) Percentage change in solvent-accessible surface area (SASA) of PTP after the addition of PEG400, CrEL, and T80. (**e**) Snapshots from a computer simulation showing the combination of 495 PEG400 molecules with 50 PTP molecules over time. (**f**) Data from isothermal titration calorimetry showing the heat changes when PEG400 is mixed with PTP; (top) raw data showing heat changes during the mixing process, and (bottom) processed data showing the integrated heat peaks against the molar ratio of PEG400 to PTP. (**g**) Photographs and Cryo-TEM images of a solution of PEG400 alone (260.0 mg·mL^–1^) and a mixture of PTP and PEG400 (PTP@PEG; 5.0 mg·mL^–1^ PTP and 260.0 mg·mL^–1^ PEG400). (**h**) Illustration of hydrogen bonds formed among PTP and PEG400 molecules. (**i**) Average lifetime of hydrogen bonding between PEG400 and two different regions of PTP: PA and PTX’. (**j**) Schematic diagram illustrating the spherical nanoparticle structures formed by PTP@PEG. (**k**) Degradation rates of PTP alone and PTP@PEG.

To further understand how PEG400 interacts with PTP, we used molecular dynamics simulations to visualize their combined assembly. The simulation revealed that PEG400 and PTP formed complexes, essentially merging into combined structures (called coassemblies) within 150 ns, with PEG400 covering on their surface (Figure 3e). To understand the force driving this combination, we measured the heat changes during the mixing process using ITC. The enthalpy change (Δ*H*) was measured to be 0.5 kJ·mol^–1^ (Figure 3f), indicating a minimal enthalpic contribution. However, the Gibbs free energy change (Δ*G* = −27.2 kJ·mol^–1^) and the positive entropy change (Δ*S* = 92.7 J·mol^–1^·K^–1^) suggested that the coassembly was strongly entropy-driven (Figure 3f). It indicates that the combination of PTP and PEG400 into the PTP@PEG coassembly is a spontaneous, meaning it occurs naturally and is favored due to an increase in disorder.

To create a combined PTP and PEG system, we dissolved PTP into a PEG400 solution, resulting in a mixture containing 260.0 mg·mL^–1^ of PEG400 and 5.0 mg·mL^–1^ of PTP (Figure 3g). This mixture, referred to as PTP@PEG, appeared clear and transparent. In contrast, a mixture of just the drug PTX and PEG400 at the same concentration, called PTX@PEG, contained a significant amount of undissolved solid material (Figure S18). Microscope examination using Cryo-TEM revealed that PTP@PEG formed tiny, spherical structures, or nanoparticles (Figure 3g). As anticipated, hydrogen bonds were formed between PEG400 and two distinct regions of the PTP molecule: the conjugated PA and a segment PTX’ (Figure 3h). The hydrogen bonds between PEG400 and PA were stronger, lasting 2.4 times longer than those between PEG400 and the PTX’ segment (46.5 ps for PA–PEG versus 19.4 ps for PTX’-PEG) (Figure 3i). These findings indicate that PEG400 primarily influences the assembly process of PTP by forming strong hydrogen bonds, particularly with the PA. Based on computer simulations and Cryo-TEM images, we believe that PEG400 surrounds the PTP molecules, causing their original fiber-like shape to transform into these nanoparticles (Figure 3j).

Changes in assembled structure—such as converting a right-handed, antiparallel duplex into left-handed, parallel-stranded switchback DNA that resists nuclease degradation—are known to affect the degradation of assemblies.^62^ To verify this, we studied the degradation behavior of PTP, both alone and in the presence of PEG400, across a range of concentrations (from 10.0 μg·mL^–1^ to 5.0 mg·mL^–1^). When PEG400 was present, the rate at which PTP broke down remained relatively constant at low concentrations (1.2 day^–1^, 1.2 day^–1^, and 1.3 day^–1^ at 10.0 μg·mL^–1^, 50.0 μg·mL^–1^, and 100.0 μg·mL^–1^, respectively). However, as the concentration of PTP increased further (0.2 mg·mL^–1^ to 5.0 mg·mL^–1^), the breakdown rate decreased from 1.1 day^–1^ to 0.4 day^–1^ (Figures 3k and S11). A similar pattern was observed when PTP was tested alone, although the rates were generally lower (Figures 3k and S11). At the highest concentration tested (5.0 mg·mL^–1^), the addition of PEG400 doubled the breakdown rate of PTP compared to when PTP alone. This suggests that in the combined PEP@PEG system, the parts of the PTP molecules susceptible to breakdown, specifically the ester bonds, are more exposed to water. Molecular dynamics simulations also showed a marked increase in ester bond SASA, indicating that PEG400 changed nanofiber packing and enhanced water accessibility around hydrolytic sites (Figure S19). In conclusion, the presence of PEG400, by preventing PTP from forming its typical assembly, changes its structure into spherical nanoparticles, consequently influencing its breakdown rate.

Sustained-release drug formulations offered benefits like less frequent dosing and more constant plasma drug levels.^63^ To explore how to achieve sustained release of PTP, we used computer simulations to study its interactions with three gel-forming substances: HA, P188, and CS (Figures 4a and S20). These substances were chosen because they are commonly used in sustained-release formulations. Our simulations focused on the hydrogen bonds formed between PTP and each substance, specifically their duration and quantity. We found that the hydrogen bonding between PTP and HA lasted significantly longer (50.3 ps) compared to those with P188 (21.2 ps) or CS (24.9 ps) (Figure 4b). While HA formed a moderate number of hydrogen bonds with PTP (75.1), it was significantly more than P188 (5.5) but less than CS (135.0) (Figures 4c and S21). Furthermore, we examined how much each substance altered the surface area of PTP accessible to the surrounding solvent (ΔSASA). HA had the largest impact (11.9%), followed by CS (10.2%), and then P188 (0.9%) (Figures 4d and S22). A larger ΔSASA in this context suggested that HA allows PTP to maintain a more aggregated, or clustered, state. Since a more aggregated form of PTP is desirable for sustained drug release, these findings suggest that HA is the most promising of the three substances for achieving sustained drug release.

**Figure 4:**
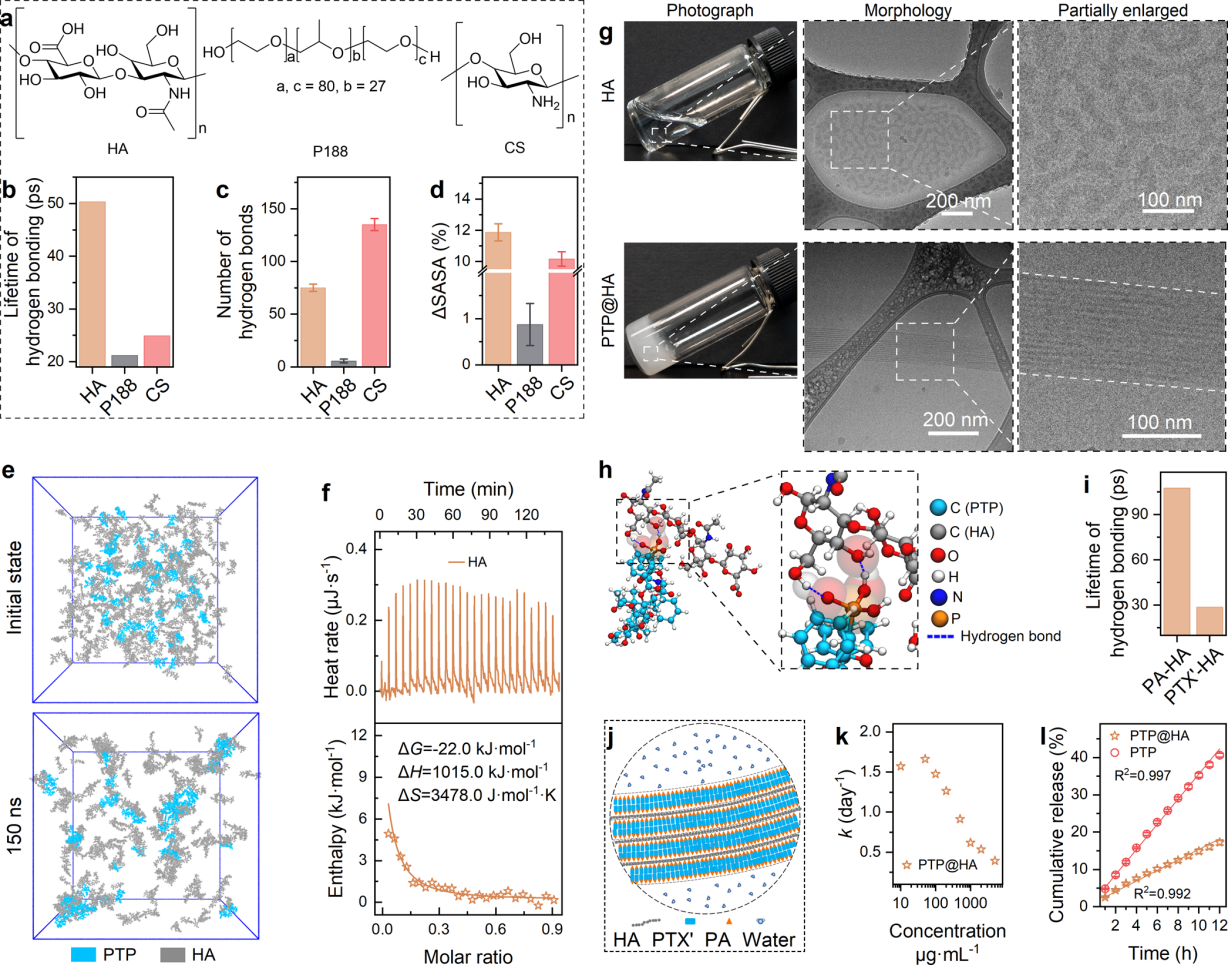
Transformation of PTP morphology into bundled fibers by HA. (**a**) The structures of HA, P188, and CS. (**b** and **c**) Average lifetime of hydrogen bonding (b) and number of hydrogen bonds (c) between PTP and each of the following: HA, P188, and CS. (**d**) Percentage change in solvent-accessible surface area (SASA) of PTP after the addition of HA, P188, or CS. (**e**) Snapshots from a computer simulation showing the combination of 250 HA molecules with 50 PTP molecules over time. (**f**) Data from ITC showing the heat changes when HA is mixed with PTP. (**g**) Photographs and Cryo-TEM images of an HA solution alone (20 mg·mL^–1^) and a mixture of PTP and HA (PTP@HA; 5.0 mg·mL^–1^ PTP and 20 mg·mL^–1^ HA). (**h**) Illustration of hydrogen bonding between PTP and HA molecules. (**i**) Average lifetime of hydrogen bonding between HA and two different regions of PTP: the PA region and the PTX’ region. (**j**) Schematic diagram illustrating the bundled fiber structures formed by the coassembly of HA and PTP. (**k**) Degradation rate of the PTP@HA at various concentrations. (**l**) Release profiles of PTP from PTP@HA (5.0 mg·mL^–1^ PTP and 20 mg·mL^–1^ HA) and PTP alone (5.0 mg·mL^–1^).

To visualize how HA interacted with PTP, we used computer simulations to track their combined assembly. Within 150 ns, PTP and HA formed multiple combined structures, or coassemblies, where HA molecules surrounded PTP (Figure 4e). To understand the forces driving the interaction between HA and PTP, we measured the heat changes during their mixing using ITC. The PTP@HA system exhibited a significantly higher enthalpy change (Δ*H* = 1015.0 kJ·mol^–1^) and a large positive entropy change (Δ*S* = 3478.0 J·mol^–1^·K^–1^) compared to the PTP@PEG system, with a Δ*G* of −22.0 kJ·mol^–1^ (Figure 4f). These values suggested a cooperatively driven process with both strong enthalpic and entropic contributions, indicative of extensive hydrogen bonding networks and molecular ordering within the bundled fibers (PTP@HA). The large enthalpic gain likely arose from multiple interaction sites between HA and PTP molecules, which facilitated a more complex and favorable assembly pathway.

We then created a mixture, termed PTP@HA, by adding PTP to a HA solution, resulting in a final concentration of 20.0 mg·mL^–1^ HA and 5.0 mg·mL^–1^ PTP (Figure 4g). This PTP@HA system exhibited a cloudy, gel-like appearance, whereas HA alone was clear and transparent. In contrast, a mixture of just the drug PTX and HA at the same concentrations, called PTX@HA, contained many solid undissolved pieces (Figure S23). Further investigation using Cryo-TEM imaging revealed that PTP@HA formed bundled fibers structures, while HA alone showed no such structures (Figure 4g). As expected, hydrogen bonds formed between HA and two specific regions of the PTP molecule: PA and the PTX’ segment (Figure 4h). Notably, the hydrogen bonds between HA and PA of PTP were significantly stronger, lasing 3.8 times longer than those between HA and PTX’ (107.7 ps for HA-PA versus 28.5 ps for PTX’-PA) (Figure 4i), highlighting the importance of the PA-HA interaction in the formation of these coassemblies. Based on the results, we believe that PTP and HA coassembled into bundled fibers with diameters greater than 100 nm and lengths extending to the micrometer (Figures 4j and S24). So, we propose that the numerous hydrogen bonds between the PA region of PTP and HA cause HA to link the PTP-formed nanofibers together, creating these large, bundled structures. The phosphate group in PTP likely plays a crucial role in this interaction.

We also studied how the presence of HA affected the degradation behavior of PTP in the PTP@HA system. At lower concentrations (10.0 μg·mL^–1^ and 50.0 μg·mL^–1^), the degradation rates were similar (1.6 and 1.7 day^–1^, respectively). However, as the concentration increased from 0.1 mg·mL^–1^ to 5.0 mg·mL^–1^, the degradation rate decreased from 1.5 day^–1^ to 0.4 day^–1^ (Figures 4k and S25). This pattern—an initial period of relatively stable degradation followed by a decrease—was similar to what was observed when PTP was tested alone. Simulations also revealed a greater SASA increase (Figure S19), suggesting HA interacted more extensively with PTP, changing the assembly structure and further exposing ester bonds to aqueous surroundings, thereby promoting degradation. Across all concentrations tested, the addition of HA increased the degradation rate of PTP. At the highest concentration (5.0 mg·mL^–1^), the degradation rate in the presence of HA was double that of PTP alone. This is likely because the combination with HA, a water-loving substance, makes PTP more exposed to water, increasing its susceptibility to degradation by hydrolysis. Finally, we examined the release of PTP from PTP@HA over time. The PTP@HA released PTP in a roughly linear fashion over 12 h, with a total release of 17.2% (Figure 4l) and an average release rate of 6.8 μg·h^–1^ (data not shown). In contrast, when PTP was tested alone, it showed a much higher total release of 40.6% (Figure 4l) and a faster average release rate of 17.5 μg·h^–1^ (data not shown). These results demonstrate that the combination of PTP and HA into bundled fiber structures in the PTP@HA effectively slows down the release rate of PTP.

While HA accelerates the hydrolysis of the PTP ester bond (Figure 4k), the release of both PTP and its hydrolysis product PTX from the PTP@HA bundled fibers is slower than from HA-free fibers (Figure 4l). Cryo-TEM (Figure 4g) and MD simulations (Figure 4i) indicate that HA cross-links adjacent nanofibers into micron-scale bundles, generating a dense hydrogel whose mesh size imposes a significant diffusion barrier. Consequently, the overall release process might be governed by a “reaction-then-diffusion” sequence in which the rate-determining step shifts from ester hydrolysis to Fickian diffusion through the bundled-fiber network.

Both PEG400 and HA are rich in oxygen atoms and hydroxyl groups, which allows them to form hydrogen bonds with PTP molecules. When either PEG400 or HA was added, the hydrogen bonds that normally form within PTP were replaced by hydrogen bonds between PTP and the added substance. In the case of PEG400, this interaction led to the formation of spherical nanoparticles, a combination called PTP@PEG. With HA, the interaction caused the PTP nanofibers to link together into bundles, a combination called PTP@HA. Therefore, through the formation of hydrogen bonds, both PEG400 and HA were able to successfully alter the original structure PTP assembly.

### Morphology-Controlled PTP Delivery Enhances *In Vivo* Antitumor Efficacy

The morphology of drug delivery systems critically affects drug transport and release, thereby tailoring their pharmacokinetic behavior and enabling the treatment of various diseases. The choice of *in vivo* models was governed by the distinct administration routes dictated by each morphology. PTP@PEG forms PEGylated nanoparticles that are fully compatible with intravenous infusion and exploit the enhanced-permeability-and-retention effect; hence the subcutaneous 4T1 model—an aggressively vascularized solid tumor—was selected to benchmark systemic antitumor performance. By contrast, PTP@HA yields a viscous hydrogel of bundled fibers (about 100 nm diameter) that cannot be injected intravenously but excels as a local depot. We therefore employed an intraperitoneal colorectal-cancer metastasis model in which sustained locoregional chemotherapy is clinically relevant. This section details the *in vivo* evaluation of PTP coassemblies with distinct morphologies—PTP@PEG nanoparticles (administered intravenously) and PTP@HA bundled fibers (administered intraperitoneally)—to assess their respective antitumor efficacy and safety.

To directly test whether the PEG400 corona stabilized PTP against hydrolysis, we incubated PTP@PEG (100 μg·mL^–1^ PTP-equivalent) and free PTP in freshly isolated rat plasma at 37 °C. PTP@PEG degraded with a rate constant of 0.19 h^–1^, whereas the free drug degraded 1.2-fold faster (*k* = 0.23 h^–1^), leaving 15.4% versus 10.4% parent compound after 10 h (Figure S26). The data confirmed that PEG400 provided effective shielding, fully consistent with the nanoparticle morphology observed in Figure 3j and providing a quantitative basis for the pharmacokinetic benefits described below.

PTP@PEG nanoparticles were designed for intravenous delivery, and their antitumor efficacy was evaluated in a subcutaneous 4T1 tumor model. PTP exhibited a wider therapeutic window than PTX. No fatalities were observed in mice treated with PTP at 100 mg·kg^–1^ (3.3 times the PTX dose), whereas PTX administration at 33.0 mg·kg^–1^ resulted in 83.3% survival after 14 days (Figure 5a). *In vitro*, PTP degraded rapidly in plasma (approximately 90% within 10 h) (Figure 5b). *In vivo* studies revealed a significant impact of PEG400 on the pharmacokinetic profile of PTP (Figure 5c). Specifically, PEG400 significantly (*p* < 0.001) increased the area under the concentration–time curve (AUC) for PTP (including its degradation product, PTX) from 1054.3 nmol·L^–1^·h (PTP group) to 2481.8 nmol·L^–1^·h (PTP@PEG group), surpassing even the PTX control (1101.0 nmol·L^–1^·h) (Figure 5d). Moreover, PEG400 also prolonged the mean residence time (MRT) and half-life (*t*_1/2_) of PTP to 0.5 and 0.8 h, respectively, which were significantly higher (*p* < 0.001) than those in the PTP group (Figure 5e). These findings demonstrate that PEG400 enhances PTP’s plasma concentration and circulation time.

**Figure 5:**
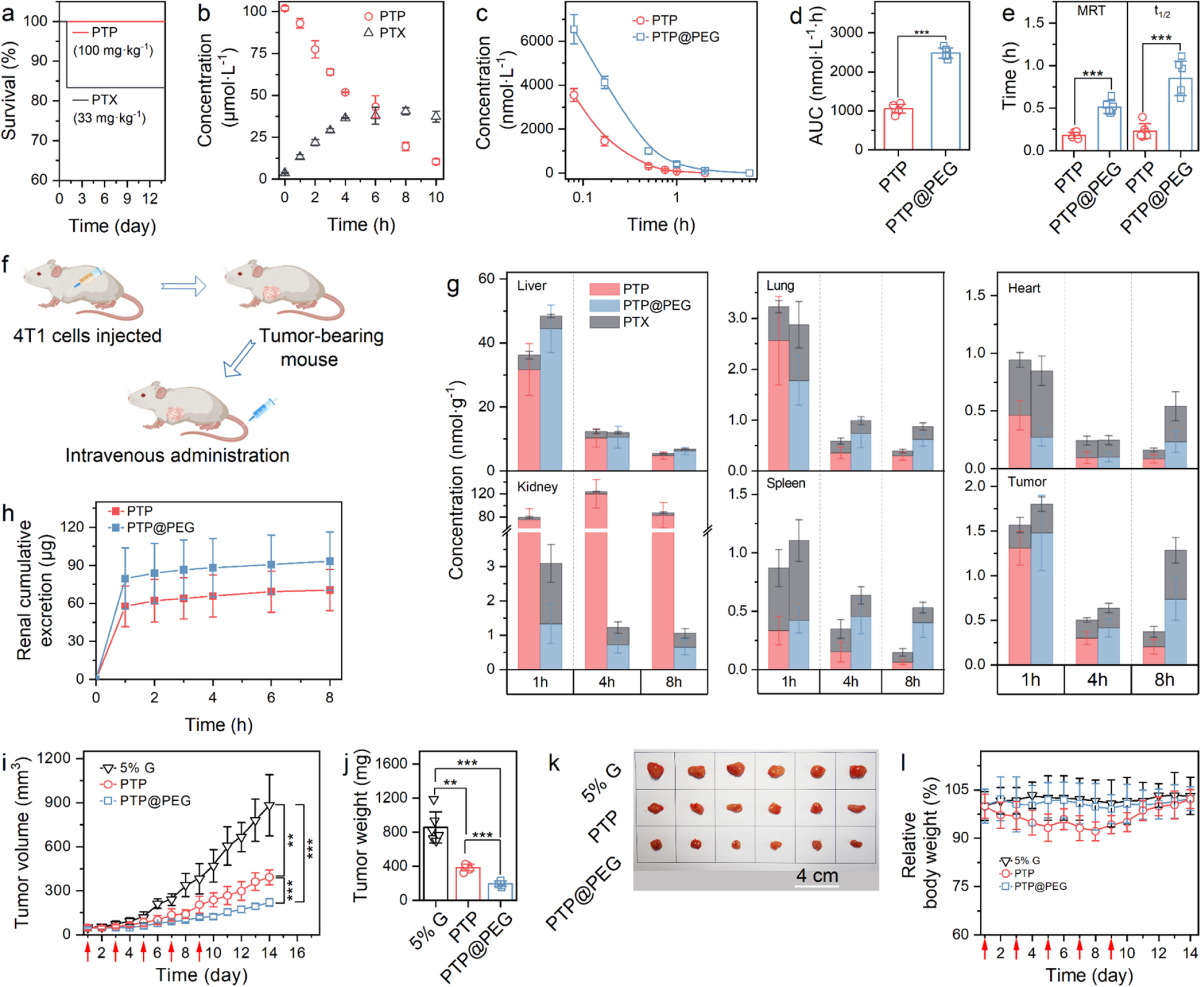
Enhanced antitumor efficacy and safety of PTP@PEG. (**a**) Survival rates of mice after administration of PTP (100.0 mg·kg^–1^) and PTX (30.0 mg·kg^–1^). (**b**) *In vitro* degradation of PTP (100.0 μg·mL^–1^) in plasma. (**c-e**) *In vivo* pharmacokinetics characteristics: pharmacokinetic curves (c), AUC (d), MRT, and *t*_1/2_ (e) for PTP and PTP@PEG. (**f**) Schematic illustration of the *in vivo* experiment. (**g**) Biodistribution of PTP and PTP@PEG both at 10.0 mg·kg^–1^ in major organs and tumor tissue postinjection. (**h**) Renal cumulative excretion of PTP and PTP@PEG both at 3.0 mg·kg^–1^. (**i-k**) *In vivo* antitumor efficacy of PTP and PTP@PEG both at 10.0 mg·kg^–1^: Average tumor volumes over time (red arrows indicate administration) (i), tumor weight (j), and photograph of tumors (k) excised from mice. (**l**) Relative body weight changes throughout the experiment. Where PTP dose is mentioned, it is equivalent to the specified PTX dose (**p < 0.01 and ***p < 0.001).

To evaluate the influence of PEG400 on PTP biodistribution, we administered PTP and PTP@PEG intravenously to 4T1 tumor-bearing mice (Figure 5f). Importantly, PTP@PEG displayed elevated concentration and slower clearance in tumor tissue, indicating enhanced therapeutic potential, possibly due to the enhanced permeability and retention effect (Figure 5g). In the PTP group, biodistribution analysis showed that PTP primarily accumulated in the liver and kidney. At 1-, 4-, and 8-h postadministration, PTP concentrations in the PTP@PEG group’s kidneys were only 3.9%, 1.0%, and 1.3%, respectively, of those in the PTP group. Moreover, PEG400 accelerated renal elimination of PTP and mitigated acute renal stress, as evidenced by the absence of hematuria (Figure 5h). The reduced kidney accumulation and accelerated renal elimination of PTP@PEG was likely attributed to the PEG coating, which could disrupt PTP’s nanofiber morphology, thereby avoiding renal trapping by the mesangium. Integration of the plasma stability and biodistribution data revealed a dual protective role of PEG400: (i) the PEG400 corona reduced renal accumulation, and (ii) it slowed systemic clearance (Figure 5g,h). These combined effects account for the observed 2.4-fold increase in AUC (Figure 5).

PTP@PEG demonstrated a superior antitumor efficacy compared to PTP *in vivo*, as evidenced by reduced tumor growth (Figures 5i and S27) and tumor weight (Figure 5j). Tumor photographs also confirmed this enhanced efficacy (Figure 5k). PTP@PEG exhibited a favorable safety profile, with no evidence of weight loss or renal damage, whereas the PTP group showed renal inflammation (Figures 5l and S28). Blood urea nitrogen (BUN) and creatinine (CREA) levels were comparable between PTP and PTP@PEG groups, suggesting potential recovery from mild renal insult over time (Figure S29). Importantly, a separate study confirmed that PEG400 potentiated PTP’s therapeutic action, as PTX and PTP exhibited similar antitumor effects (Figure S30).

PTP@HA bundled fibers were designed for intraperitoneal administration, and their antitumor efficacy was evaluated in a colorectal cancer peritoneal metastasis model. Because it was difficult to distinguish between free PTP and PTP retained within the PTP@HA fibers, we measured the PTP blood concentrations over time to indicate the drug release profile from PTP@HA. Following PTP@HA administration, PTP concentration initially increased and then decreased (Figure 6a). The MRT of PTP in the PTP@HA group (83.9 h) was 19.1 times that of the PTX group (4.4 h), and the *t*_1/2_ of PTP@HA (76.7 h) was 24.0 times that of PTX (3.2 h) (Figure 6b). These results indicated that the PTP@HA fibers prolonged the retention of PTP in the peritoneal cavity.

**Figure 6:**
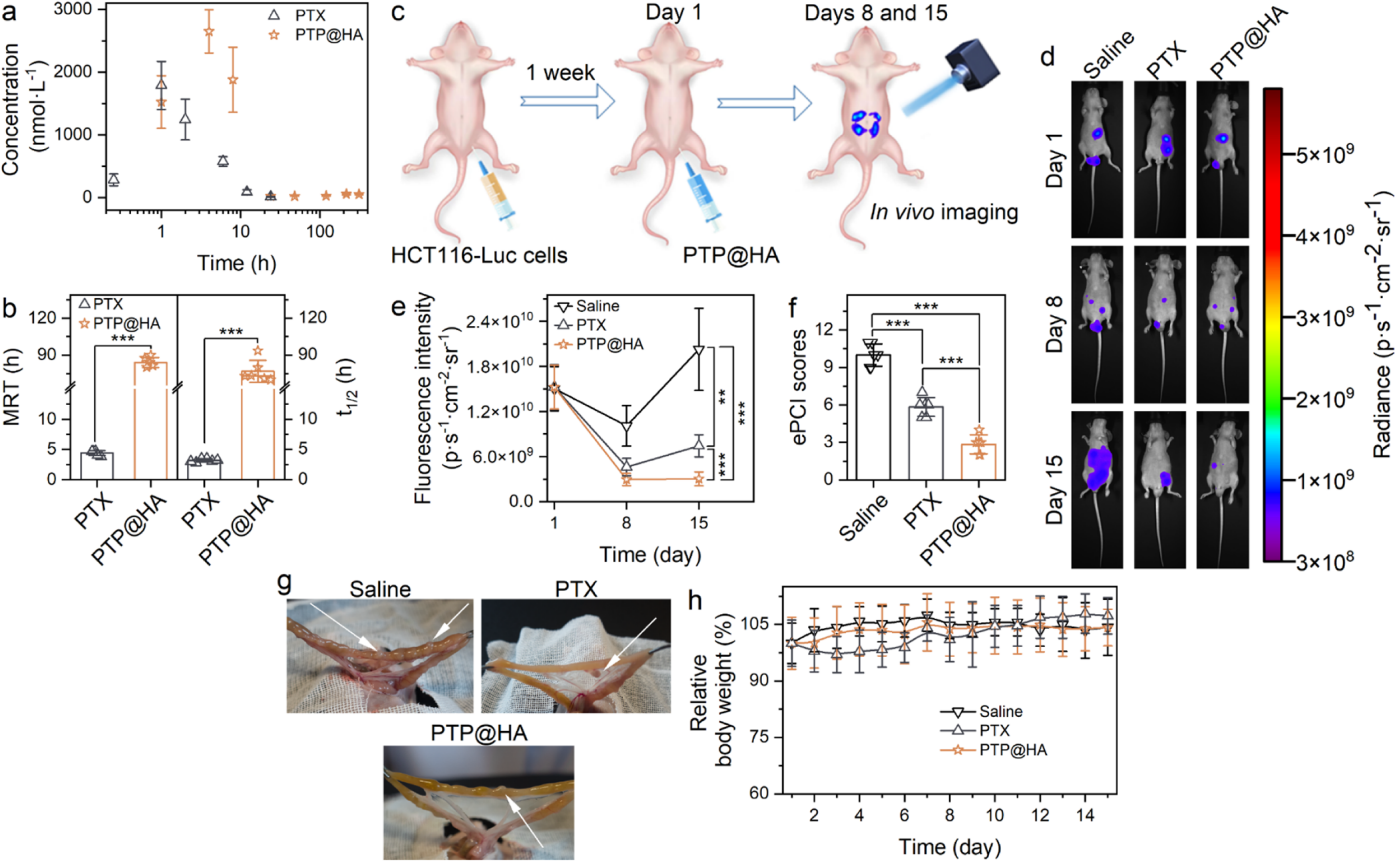
PTP@HA enhanced antitumor efficacy in a colorectal cancer peritoneal metastasis model. (**a** and **b**) *In vivo* pharmacokinetic characteristics: PTP concentration–time curves (a), MRT and *t*_1/2_ (b) of PTP@HA (100.0 mg·kg^–1^) and PTX solution (12.0 mg·kg^–1^). (**c**) Schematic illustration of the *in vivo* experimental procedure. Both PTX solution and PTP@HA delivered a 45.0 mg·kg^–1^ PTX equivalent dose, with PTX solution administered three times while PTP@HA was just once. (**d** and **e**) Representative *in vivo* fluorescence images (d) and fluorescence intensity of tumors (e) with treatments over 15 days. (**f** and **g**) ePCI scores (g) and representative photographs (g) of tumors in mice on day 15 post-treatment. The white arrows indicated the location of tumors. (**h**) Relative body weight changes in mice throughout the experiment (**p < 0.01 and ***p < 0.001).

To assess the antitumor effects of PTP@HA, we established a colorectal cancer peritoneal metastasis model (Figure 6c).^64^ Nude mice were injected intraperitoneally with HCT116-Luc cells, and tumor growth was monitored by fluorescence imaging of the resulting luciferase activity (Figure 6d). One week after tumor cell injection, the mice with established tumors were treated. Highlighting the advantage of its sustained-release formulation, the PTP@HA group required only a single intraperitoneal injection delivering 45.0 mg·kg^–1^ PTX equivalent (in a low volume of 9.0 μL·g^–1^). In contrast, the conventional PTX solution group needed three separate injections (15.0 mg·kg^–1^ PTX per injection; 25.0 μL·g^–1^ each) at 4-h intervals to deliver the same total dose of 45.0 mg·kg^–1^ PTX. This multidose approach for the PTX solution was primarily due to the drug’s limited solubility, which prevents delivering the total dose in a single, reasonably sized injection volume. Moreover, splitting the dose helps mitigate the acute toxicity risk linked to the high peak concentrations caused by PTX’s rapid, uncontrolled release from a simple solution.

On the first day after treatment, the PTP@HA group exhibited a fluorescence intensity of 1.5 × 10^10^ p·s^–1^·cm^–2^·sr^–1^, which decreased substantially to 3.04 × 10^9^ p·s^–1^·cm^–2^·sr^–1^ by day 8 post-treatment (Figure 6e). By day 15, the fluorescence in the PTP@HA group (2.95 × 10^9^ p·s^–1^·cm^–2^·sr^–1^) remained significantly lower (*p* < 0.001) compared to the control group receiving saline (Figure 6e). Crucially, despite the single administration, the fluorescence in the PTP@HA group was only 0.7-fold and 0.4-fold that of the multi-injection PTX-treated group on days 8 and 15, respectively (Figure 6e), demonstrating progressively superior efficacy over time.

On day 15, nude mice were euthanized, and their abdominal cavities were examined. The observed tumor growth, along with significantly lower experimental peritoneal carcinomatosis index (ePCI) scores (Figure 6f) and photographs of the tumors (Figure 6g), confirmed that single-dose PTP@HA was more effective in inhibiting tumor progression compared to both saline and the multidose PTX solution regimen. Furthermore, PTP@HA demonstrated a favorable safety profile, as evidenced by the lack of significant changes in body weight throughout the study (Figure 6h). These results underscore the therapeutic benefit derived from the sustained intraperitoneal drug release facilitated by the PTP@HA hydrogel formulation, which allows for a simplified, single-injection administration while overcoming the solubility limitations and mitigating the toxicity risks associated with the uncontrolled release kinetics of conventional PTX solutions.

Collectively, these results underscore the critical role of PEG400 and HA in altering PTP’s assembly morphology via hydrogen bonding. Specifically, intravenous injection of PTP@PEG nanoparticles allowed at least 95.0% of the PTP, previously accumulated in the kidneys, to enter the bloodstream. This increased the drug concentration, prolonged its elimination half-life, and further boosted its antitumor activity. Additionally, PTP@HA bundled fibers, with sustained release properties, directly interacted with the tumor in the peritoneal cavity, increasing contact duration and further enhancing the antitumor effect. Thus, by altering morphology, PTP was made suitable for both intravenous and intraperitoneal administration.

## Conclusions

The rational design of drug nanocarriers is a central goal in advanced drug delivery, as their morphology governs pharmacokinetic fate and, consequently, therapeutic efficacy.^24,25,27,29^ However, achieving robust and tunable control over morphology, especially from a single molecular entity, remains a formidable challenge.^20,43^ Here, we report a powerful and versatile strategy that leverages hydrogen bonding to program the self-assembly of a novel paclitaxel derivative, PTP, into distinct, functionally optimized nanostructures. By introducing a phosphate group onto the paclitaxel scaffold, we created an amphiphile that spontaneously forms uniform nanofibers in an aqueous environment, driven by strong, directional hydrogen bonds. More importantly, we showed that this foundational nanofiber morphology could be rationally transformed—either disassembled into spherical nanoparticles with PEG400 or cross-linked into bundled fibers with HA—by selectively competing with or reinforcing these guiding hydrogen bonds. This morphological control allowed us to tailor the drug’s performance for two distinct cancer treatment scenarios, showcasing an unprecedented level of adaptability from a single derivative.

The self-assembly of PTP into nanofibers is a direct consequence of the strategically introduced phosphate group. Our molecular dynamics simulations confirmed that the hydrogen bonds formed between the phosphate moieties of adjacent PTP molecules are the primary stabilizing force, significantly stronger than the intrinsic interactions within the native PTX structure. This robust, one-dimensional assembly creates nanofibers with high aspect ratios, a morphology known to influence biological interactions. However, such fibrillar structures can also lead to suboptimal pharmacokinetics, including rapid clearance and off-target accumulation. Indeed, our *in vivo* studies revealed that the pristine PTP nanofibers, when administered intravenously, accumulated significantly in the kidneys. This renal sequestration is a known issue for certain elongated nanostructures, which can become physically entrapped within the renal mesangium, leading to potential nephrotoxicity and reducing the drug fraction available for therapeutic action at the tumor site.^38,65^

Our key innovation was the strategic manipulation of this nanofiber assembly using common, biocompatible excipients. The coassembly of PTP with PEG400 resulted in a dramatic morphological shift from nanofibers to discrete, spherical nanoparticles (PTP@PEG). The mechanism, as supported by our simulations and ITC data, involves the formation of competing hydrogen bonds between the abundant hydroxyl groups of PEG400 and the phosphate groups of PTP. This interaction effectively “coats” the PTP molecules, disrupting the intermolecular PTP–PTP hydrogen bonding network required for fiber elongation and instead promoting the formation of compact, PEGylated nanospheres.

This morphology-switching had profound and beneficial pharmacological consequences. The transformation into small nanoparticles prevented renal trapping,^38^ evidenced by a remarkable >95% reduction in kidney accumulation compared to the PTP nanofiber group. Simultaneously, the PEGylated surface provided a “stealth” effect, significantly extending the drug’s circulation half-life and increasing the overall systemic exposure (AUC). This enhanced pharmacokinetic profile translated directly to superior antitumor efficacy in the 4T1 breast cancer model, as the prolonged circulation and reduced renal clearance allowed for greater accumulation of the drug in the tumor tissue, likely via the enhanced permeability and retention effect. This result elegantly demonstrates how targeted disruption of a self-assembly pathway can be used to solve a specific drug delivery challenge like nephrotoxicity.

In a complementary approach, we demonstrated that the PTP nanofibers could be reinforced, rather than disrupted, to create a system suitable for local, sustained-release therapy. Coassembly with HA, a natural biopolymer rich in hydrogen-bond donors and acceptors, did not disassemble the PTP nanofibers but instead acted as a multivalent “glue,” cross-linking them into thick, bundled fibers (PTP@HA). This process resulted in the formation of a viscous hydrogel-like depot. When administered intraperitoneally, this PTP@HA formulation provided highly effective, sustained drug release directly within the peritoneal cavity, the site of colorectal cancer metastasis in our model. The pharmacokinetic data confirmed this mechanism, showing a 24-fold longer half-life for PTP@HA compared to the conventional PTX solution. This sustained local concentration enabled a superior therapeutic outcome with a single administration, outperforming a multidose regimen of PTX solution while improving safety and simplifying the treatment schedule. This finding highlights the versatility of our platform, where reinforcing the primary hydrogen bonds leads to a completely different, yet equally valuable, therapeutic application.

It is important to contrast our approach with conventional paclitaxel formulations like Taxol, which relies on the surfactant Cremophor EL.^61^ While effective at solubilizing PTX, Cremophor EL is associated with significant toxicities, including hypersensitivity reactions, and does not offer morphological control. Our system not only avoids such toxic excipients but provides a tunable platform where the drug itself is the primary component of the nanostructure. By modifying the drug, we unlock the potential for self-assembly, and by using well-established, safe excipients like PEG and HA as “morphology modifiers,” we can direct that assembly toward a desired therapeutic outcome.

Beyond the therapeutic advantages, our platform’s design facilitates its readiness for clinical translation. The preparation method is remarkably straightforward, requiring only simple mixing of the drug derivative with common, biocompatible excipients like PEG400 and HA—both of which have a long history of clinical use and well-established safety profiles. This stands in stark contrast to many complex nanomedicine manufacturing techniques that rely on sophisticated equipment and stringent process controls.^66^ Our “bottom-up”, self-assembly approach is inherently simpler, more scalable, and less dependent on complex machinery, which not only lowers potential manufacturing costs but also simplifies process validation and adherence to Good Manufacturing Practice standards.^43,66^ The combination of a simple, robust formulation process with clinically established excipients significantly derisks the development pathway, paving a clearer and more direct route from the laboratory to potential clinical applications.^67^

While this work establishes a strong proof-of-concept, future studies could explore the full potential of this hydrogen-bond-guided platform, particularly for applications in glioma^68^ and spinal cord injury.^69,70^ Long-term toxicity studies of PTP and its coassemblies, particularly with repeated dosing of PTP@PEG, are necessary for clinical translation, even though PEG400 and HA are generally considered safe and acute toxicity profiles were favorable. Furthermore, the generalizability of this hydrogen-bond-guided strategy to other classes of poorly soluble drugs represents an exciting frontier for developing next-generation, morphologically tunable drug delivery systems. Investigating whether these engineered nanostructures can overcome known mechanisms of drug resistance would also be of significant clinical interest.

The synthesis of PTP is straightforward, involving six steps with chromatographic purification required only up to step three. This synthetic approach has been successfully scaled to 5 g batches without yield reduction. The proposed clinical formulations—PTP@PEG injection and PTP@HA depot—are prepared through simple solution or dispersion mixing, operations routinely validated under current Good Manufacturing Practice standards. The excipients employed, PEG400 and hyaluronic acid, comply with both United States Pharmacopeia and European Pharmacopoeia standards, confirming their established safety profile for pharmaceutical use and eliminating the need for additional toxicological evaluations.

From a regulatory perspective, PTP would be classified as a new chemical entity, necessitating a comprehensive Chemistry, Manufacturing, and Controls dossier as well as thorough safety assessments. However, existing toxicological data for paclitaxel may be leveraged through the US Food and Drug Administration’s 505­(b)(2) regulatory pathway, allowing reference to previously established drug safety information. Overall, these considerations suggest relatively low technical and regulatory hurdles for transitioning PTP from laboratory-scale production to commercial manufacturing and clinical application.

In summary, this study demonstrates that the rational design and manipulation of hydrogen bonds is a powerful strategy for engineering drug delivery systems. We successfully engineered a paclitaxel derivative, PTP, whose phosphate group directs robust hydrogen-bond-driven self-assembly into nanofibers. Through strategic coassembly, these nanofibers were transformed into distinct morphologies: spherical nanoparticles (PTP@PEG) via hydrogen bond competition with PEG400, and bundled fibers (PTP@HA) via hydrogen bond reinforcement with HA. These tailored nanostructures exhibited profoundly different pharmacokinetic profiles and therapeutic outcomes. Intravenously administered PTP@PEG nanoparticles significantly reduced renal PTP accumulation (to approximately 1.3% of that observed with PTP nanofibers alone), enhanced systemic circulation, and improved antitumor efficacy in a breast cancer model. Intraperitoneally administered PTP@HA bundled fibers provided sustained peritoneal drug release and demonstrated superior single-dose efficacy in a colorectal cancer peritoneal metastasis model. This work highlights hydrogen bonding as a powerful and versatile molecular design tool for the precise morphological engineering of drug assemblies. It offers a promising and adaptable paradigm for optimizing drug delivery, potentially unlocking the full therapeutic potential of various compounds by tailoring their nanostructure to meet diverse clinical needs, thereby paving the way for more effective and “programmable” drug formulations.

## Methods

### Materials and Reagents

Paclitaxel (PTX) was purchased from Wuxi Taxus Pharmaceuticals Co., Ltd. (China). PTX solution was obtained from Yangtze River Pharmaceuticals Co., Ltd. (China). 3-Phosphonopropanoic acid (PA), *N*,*N*-diisopropylethylamine (DIPEA), benzyl alcohol, 4-dimethylaminopyridine (DMAP), 1-hydroxybenzotriazole (HOBt), and Pd/C were purchased from Titan Scientific Co., Ltd. (China). Ammonium acetate was obtained from ROE Scientific Inc. (USA). Pyridine, *N*-(3-(dimethylamino)­propyl)-*N*′-ethylcarbodiimide hydrochloride (EDCI), disodium hydrogen phosphate, phosphoric acid, PEG400 and HA were purchased from Shanghai Aladdin Biochemical Technology Co., Ltd. (China). Dichloromethane (DCM), *N*,*N*-dimethylformamide (DMF) and tetrahydrofuran (THF) were obtained from Lingfeng Chemical Reagent Co., Ltd. (China). Acetonitrile was obtained from Tedia High Purity Solvents Co. Ltd. (USA). Methanol was obtained from CINC High Purity Solvents Co., Ltd. (China) for general use and from Merck KGaA (Germany) for mass spectrometry analysis. RPMI 1640 medium, DMEM medium, trypsin-EDTA solution and streptomycin were obtained from Thermo Fisher Scientific Inc. (USA). Fetal bovine serum was purchased from BioChannel Biotech Co., Ltd. (China). The mouse breast 4T1 cells, human lung A549 cells, and human ovarian SKOV3 cells were obtained from the American Type Culture Collection (USA). HCT116-Luc cells were obtained from Zhong Qiao Xin Zhou Biotech Co., Ltd. (China). CCK8 cell counting kit was obtained from Dojindo Laboratories (Japan). Tubulin protein and general tubulin buffer (PEM buffer) were obtained from Cytoskeleton, Inc. (USA). D-Luciferin potassium salt was purchased from Beyotime Biotech Inc. (China). Saline injections and 5% glucose were purchased from Shuanghe Pharmaceuticals Co., Ltd. (China). Phosphate buffer saline (PBS) was obtained from Keygen Biotech Co. Ltd. (China).

### Point Charge Derivation

Three-dimensional structures of PTX derivatives, chemically modified with R_1_-R_5_, were constructed using GaussView (Gaussian Inc., USA). Structural optimizations were then performed using Gaussian 16W (Gaussian Inc., USA) at the B3LYP-D3­(BJ)/def2-SVP level of theory. Following optimization, single-point energy calculations were conducted at the B3LYP-D3­(BJ)/def2-TZVP level to generate molecular surface electrostatic potential data. Finally, RESP2 atomic charges were derived using Multiwfn.^71^

### Molecular Dynamics Simulation General Methodology

All molecular dynamics simulations were performed using GROMACS 2019.6 ^72^ with consistent parameters unless otherwise specified in specific simulation protocols. The AMBER14SB/GAFF force field^73–75^ and the TIP3P water model^76^ were employed for all simulations. Initial structure files were generated and optimized using BIOVIA Discovery Studio 2016. Short-range van der Waals interactions were treated with a 1 nm cutoff. Long-range electrostatic interactions were calculated using the Particle Mesh Ewald method. Trajectory analysis was performed using VMD 1.9.3. Data analysis was performed by GROMACS built-in tools. All systems were confirmed to reach the steady state prior to analysis. To calculate the SASA of ester bonds on the PTP molecular surface, atomic indices of the relevant components were first identified using an index file. The SASA was then computed using VMD with a probe radius of 1.4 Å.

### Derivative Screening Simulations

For PTX derivative screening simulations, the system containing 50 derivative molecules (PTX conjugated with R_1_-R_5_) were prepared. Derivative molecules were randomly placed using Packmol^77^ and solvated with water using the “gmx solvate” module. Energy minimization was performed in two stages: 20 000 steps of steepest descent, followed by 100 000 steps of conjugate gradient. Production molecular dynamics simulations were performed in the NPT ensemble for 150 ns at 298.15 K and 1 bar pressure, with a 0.001 ps time step.

### Excipient Interaction Simulations

To investigate PTP interaction with excipients (PEG400, CrEL, T80, HA, P188, and CS), systems were prepared with 50 PTP molecules. Excipient molecules were then randomly placed using Packmol, substituting for water molecules. For high molecular weight excipients (HA, P188, and CS), polymer repeating units were included in a reasonable number to facilitate all-atom simulations. The simulation box size and related parameters remained consistent across all excipient experiments. Energy minimization and 150 ns production simulations were performed using the same protocols as in derivative screening simulations.

### PTP Fiber Structure Generation and Interaction Analysis

A cubic box (5 × 5 × 5 nm^3^) with periodic boundary was established, and four PTP molecules were randomly inserted and solvated with water. The system was initially energy minimized and then equilibrated in NVT and NPT ensembles. An annealing process was then performed, cycling the temperature from 298 to 900 K and back to 298 K every 1000 ps for 200 cycles. The lowest energy PTP tetramer structure obtained from annealing was used as a building block. Structures containing 32 PTP molecules were generated by expanding this tetramer. A subsequent 100 ns simulation was conducted at 298 K to obtain stable PTP nanofiber structures, discarding unstable structures during the simulation. The dimer structures of PTP and PTX molecules with the lowest energy were obtained through an annealing method. The IGMH analysis of the dimer structures of PTP and PTX molecules was performed using Multiwfn.^78^ Additionally, intermolecular interactions were investigated using the EDA-FF method in Multiwfn.

### Molecular Docking

The crystal structure of alpha-beta tubulin from zinc-induced sheets stabilized with PTX (PDB ID: 1JFF)^79^ was obtained from the Protein Data Bank. The molecular structures of PTP and PTX were prepared and optimized using BIOVIA Discovery Studio 2016. Protein and small molecule (PTP and PTX) structures were preprocessed using Maestro 11.9. Docking scores were then calculated using Schrödinger Release 2019-1 by docking a PTP or PTX molecule into the protein structure.^80–82^

### Synthesis and Characterization of MPA

3-Phosphonopropanoic acid (PA) (500.0 mg, 3.2 mmol, 1.0 equiv) was added to methanol (10.0 mL).^55^ The mixture was stirred for 4 days at room temperature. The solvent was then concentrated under vacuum to yield (3-methoxy-3-oxopropyl) phosphoric acid (MPA) as a white solid (528.2 mg, 3.1 mmol, 96.9% yield). ESI-MS (*m*/*z*): [M – H]^−^ found 167.0110. MPA was used directly in subsequent reactions without further purification. The purities of PA and MPA were assessed using HPLC (SPD-M20A, Shimadzu, Japan) with a Poroshell 120 HILIC-Z column (4.6 × 100 mm, 2.7 μm, Agilent, USA). The analysis employed a flow rate of 0.5 mL·min^–1^ and a sample injection volume of 3.0 μL. Column temperature and detection wavelength were set at 25 °C and 206 nm, respectively. The mobile phase consisted of acetonitrile and a 0.01 mol·L^–1^ disodium hydrogen phosphate solution (pH adjusted to 6.7 with phosphoric acid) in a constant ratio of 7:3 (v/v).

### Synthesis and Characterization of MBPA

MPA (528.2 mg) sulfinyl chloride solution (5.0 mL) was treated with DMF (5 drops), stirred at 60 °C for 1 h, and then concentrated under vacuum to yield the pale-yellow liquid intermediate (presumed MPA-Cl).^55^ This intermediate was dissolved in DCM (12.0 mL) and cooled in an ice bath for 10 min. Separately prepared solution A (256.7 mg pyridine in 6.0 mL DCM) and solution B (2516.7 mg DIPEA and 877.4 mg benzyl alcohol in 15.0 mL DCM) were then sequentially added to the cooled intermediate solution. The reaction was then warmed to room temperature and stirred overnight. Following the reaction, the mixture was diluted with DCM (7.0 mL) and washed sequentially with 0.1 M HCl (50 mL × 3) and water (50 mL × 1); the organic phase was subsequently concentrated under vacuum.^83^ The crude product was purified by silica gel column chromatography using an eluent of CHCl_2_:MeOH (100:1) to yield methyl 3-(bis­(benzyloxy)­phosphoryl)­propanoate (MBPA) as a pale yellow liquid (836.0 mg, 77.4% yield). Characterization data: ^1^H NMR (500 MHz, DMSO-*d*_6_) δ 7.46–7.27 (m, 10H), 5.09–4.89 (m, 4H), 3.55 (s, 3H), 2.50–2.43 (m, 2H), 2.15–2.05 (m, 2H); ESI-MS (*m*/*z*): [M + Na]^+^ found 371.1017.

### Synthesis and Characterization of BPA

To a MBPA (836.0 mg) THF solution (30.0 mL), cooled in an ice bath, was added 1 M NaOH (3.0 mL). The mixture was warmed to room temperature and stirred for 2.5 h.^55^ The reaction was quenched with water (30.0 mL) and then vacuum-concentrated to remove THF. The resulting mixture was extracted with DCM (15.0 mL × 2). The aqueous phase was acidified to pH = 1 using 0.5 M HCl and subsequently extracted with DCM (50.0 mL × 1). The final organic extract was washed with water and concentrated under vacuum. This procedure yielded 3-(bis­(benzyloxy)­phosphoryl)­propanoic acid (BPA) as a pale yellow waxy solid (660.2 mg, 83.3% yield) with >97% HPLC purity (Figure S31). ^1^H NMR (500 MHz, CDCl_3_) δ 7.39–7.27 (m, 10H), 5.04 (dd, J = 11.8 Hz, 9.0 Hz, 2H), 4.96 (dd, J = 11.8 Hz, 8.1 Hz, 2H), 2.62–2.53 (m, 2H), 2.15–2.05 (m, 2H). ^13^C NMR (126 MHz, CDCl_3_) δ 175.37, 175.23, 136.06, 136.01, 128.70, 128.61, 128.05, 67.75, 67.70, 27.19, 27.16, 21.83, 20.68. ^31^P NMR (202 MHz, CDCl_3_) δ 31.67. ESI-MS (*m*/*z*): [M – H]^−^ found 333.0890.

### Synthesis and Characterization of PTX-BPA

A solution of BPA (245.0 mg) in DCM (30.0 mL) was cooled in an ice bath. Subsequently, *N*-(3-(Dimethylamino)­propyl)-*N*′-ethylcarbodiimide hydrochloride (EDCI; 174.2 mg), 4-dimethylaminopyridine (DMAP; 111.0 mg) and 1-hydroxybenzotriazole (HOBt; 90.0 mg) were sequentially added. The mixture was stirred for 3 h, after which PTX powder (517.1 mg) was added.^56^ The reaction mixture was warmed to room temperature and stirred for 6 h. The mixture was then washed sequentially, first with 0.1 M NaOH (50 mL × 1), water (50 mL × 1), 0.1 M HCl (50 mL × 1), and a final portion of water (50 mL × 1). The organic phase was concentrated under vacuum, and the crude product was purified by silica gel column chromatography using CHCl_2_:MeOH (100:1) as the eluent. This yielded PTX-2’–OOC-P-Bn (PTX-BPA) as a white solid (633.8 mg, 83.3% yield). ^1^H NMR (500 MHz, CDCl_3_) δ 8.17–8.13 (m, 2H), 7.89–7.83 (m, 3H), 7.63–7.57 (m, 1H), 7.52 (t, J = 7.7 Hz, 2H), 7.49–7.43 (m, 1H), 7.43–7.26 (m, 16H), 7.25–7.23 (m, 1H), 6.29 (s, 1H), 6.23 (t, J = 8.7 Hz, 1H), 6.01 (dd, J = 8.9 Hz, 3.3 Hz, 1H), 5.68 (d, J = 7.1 Hz, 1H), 5.45 (d, J = 3.4 Hz, 1H), 5.01–4.82 (m, 5H), 4.45 (dd, J = 11.0 Hz, 6.6 Hz, 1H), 4.31 (d, J = 8.5 Hz, 1H), 4.21 (d, J = 8.5 Hz, 1H), 3.81 (d, J = 7.0 Hz, 1H), 2.71–2.51 (m, 3H), 2.47 (s, 3H), 2.35 (dd, J = 15.4 Hz, 9.4 Hz, 1H), 2.22 (s, 3H), 2.15–1.95 (m, 3H), 1.93 (s, 3H), 1.91–1.84 (m, 1H), 1.68 (s, 3H), 1.23 (s, 3H), 1.13 (s, 3H). ^13^C NMR (126 MHz, CDCl_3_) δ 203.89, 171.27, 170.83, 170.72, 169.93, 167.92, 167.42, 167.11, 142.89, 137.29, 136.11, 136.06, 133.84, 133.71, 132.82, 131.83, 130.31, 129.29, 129.01, 128.80, 128.73, 128.70, 128.67, 128.63, 128.51, 128.36, 128.05, 127.63, 126.68, 84.52, 81.11, 79.24, 76.50, 75.67, 75.21, 74.90, 72.18, 71.86, 67.68, 67.63, 67.59, 67.54, 58.59, 53.00, 45.63, 43.23, 35.60, 27.45, 26.88, 22.80, 22.23, 21.97, 20.86, 14.84, 9.66. ^31^P NMR (202 MHz, CDCl_3_) δ 30.25. ESI-MS (*m*/*z*): found [M + H]^+^ 1170.4240, [M + Na]^+^ 1192.4059.

### Characterization of PTX

To facilitate comparison and analysis, NMR spectroscopy was performed on PTX. ^1^H NMR (500 MHz, CDCl_3_) δ 8.14–8.10 (m, 2H), 7.75–7.71 (m, 2H), 7.63–7.58 (m, 1H), 7.53–7.45 (m, 5H), 7.43–7.31 (m, 5H), 7.02 (d, *J* = 8.9 Hz, 1H), 6.27 (s, 1H), 6.25–6.19 (m, 1H), 5.78 (dd, *J* = 8.9 Hz, 2.7 Hz, 1H), 5.67 (d, *J* = 7.1 Hz, 1H), 4.93 (dd, *J* = 9.7 Hz, 2.3 Hz, 1H), 4.78 (d, *J* = 2.7 Hz, 1H), 4.39 (dd, *J* = 10.9 Hz, 6.7 Hz, 1H), 4.29 (d, *J* = 8.4 Hz, 1H), 4.19 (dd, *J* = 8.4 Hz, 1.0 Hz, 1H), 3.79 (d, *J* = 7.0 Hz, 1H), 3.64 (bs, 1H), 2.53 (ddd, *J* = 14.7 Hz, 9.7 Hz, 6.7 Hz, 1H), 2.47 (bs, 1H), 2.38 (s, 3H), 2.36–2.31 (m, 1H), 2.27 (dd, *J* = 15.5 Hz, 9.0 Hz, 1H), 2.22 (s, 3H), 1.87 (ddd, *J* = 14.7 Hz, 10.9 Hz, 2.4 Hz, 1H), 1.79 (d, *J* = 1.4 Hz, 4H), 1.68 (s, 3H), 1.23 (s, 3H), 1.14 (s, 3H). ^13^C NMR (126 MHz, CDCl_3_) δ 203.67, 172.74, 171.28, 170.42, 167.11, 167.05, 142.02, 138.06, 133.77, 133.71, 133.25, 132.00, 130.26, 129.22, 129.07, 128.78, 128.74, 128.40, 127.11, 127.10, 84.46, 81.21, 79.11, 76.56, 75.62, 75.02, 73.28, 72.37, 72.23, 58.67, 55.12, 45.71, 43.23, 35.76, 35.68, 26.92, 22.67, 21.86, 20.89, 14.87, 9.62.

### Synthesis and Characterization of PTP

PTX-BPA (512.0 mg, 0.4 mmol, 1.0 equiv) was dispersed in 50.0 mL of methanol. Subsequently, a solution of ammonium formate (414.0 mg, 6.6 mmol, 16.5 equiv) in 5.0 mL of water was added to the dispersion, followed by the addition of 10% Pd/C (102.4 mg) under an argon atmosphere.^57^ The mixture was stirred at 50 °C for 30 min, then filtered through a 0.22 μm membrane and concentrated under vacuum. The crude product was dissolved in 20.0 mL of methanol and purified via preparative-high performance liquid chromatography (pre-HPLC). The target compound PTX-2’–OOC-P (PTP) was obtained as a white solid (342.2 mg, 0.3 mmol, 75.0% yield). ^1^H NMR (400 MHz, DMSO-*d*_6_, 1d D_2_O) δ 7.96 (d, *J* = 7.6 Hz, 2H), 7.83 (d, *J* = 7.5 Hz, 2H), 7.75–7.61 (m, 3H), 7.59–7.37 (m, 7H), 6.27 (s, 1H), 5.81 (t, *J* = 9.2 Hz, 1H), 5.53–5.46 (m, 1H), 5.39 (d, *J* = 7.1 Hz, 1H), 5.30 (d, *J* = 9.0 Hz, 1H), 4.90 (d, *J* = 9.4 Hz, 1H), 4.09 (dd, *J* = 10.8 Hz, 6.9 Hz, 1H), 3.99 (s, 2H), 3.57 (d, *J* = 7.1 Hz, 1H), 2.37–2.28 (m, 1H), 2.21 (s, 3H), 2.08 (s, 3H), 1.85–1.71 (m, 4H), 1.68–1.56 (m, 3H), 1.55–1.49 (m, 1H), 1.47 (s, 3H), 0.99 (s, 3H), 0.98 (s, 3H). ^13^C NMR (126 MHz DMSO-*d*_6_) δ 202.93, 173.64, 173.49, 170.25, 169.84, 169.26, 166.99, 166.91, 165.78, 140.03, 138.23, 138.19, 134.74, 134.69, 134.00, 133.85, 131.83, 130.46, 130.08, 129.25, 129.12, 128.77, 128.57, 128.21, 128.12, 84.17, 80.78, 77.22, 75.82, 75.28, 75.03, 71.31, 70.85, 57.89, 54.62, 46.60, 43.47, 37.03, 34.94, 29.61, 26.88, 25.66, 24.58, 22.98, 21.91, 21.17, 14.45, 10.29. ^31^P NMR (202 MHz, DMSO-*d*_6_) δ 18.75. ESMS *m*/*z*: 988.3158 [M – H]^−^.

### PTP Degradation and Structural Analysis

A dispersion of 10 mg·mL^–1^ PTP (50.0 mg, 0.05 mmol, 1.0 equiv) in PBS was reacted at 50 °C for 11 h. The reaction mixture was evaporated to dryness, and the resulting residue was dissolved in a mixture of acetonitrile (3.0 mL) and water (0.8 mL). The obtained solution was purified via pre-HPLC. The degradation product PTX was obtained as a white solid (25.6 mg, 0.03 mmol, 60.0% yield). ^1^H NMR (500 MHz, CDCl_3_) δ 8.12 (d, *J* = 7.1 Hz, 2H), 7.73 (d, *J* = 7.1 Hz, 2H), 7.61 (t, *J* = 7.4 Hz, 1H), 7.55–7.45 (m, 5H), 7.44–7.30 (m, 5H), 7.03 (d, *J* = 8.9 Hz, 1H), 6.27 (s, 1H), 6.25–6.17 (m, 1H), 5.78 (dd, *J* = 8.8 Hz, 2.8 Hz, 1H), 5.67 (d, *J* = 7.0 Hz, 1H), 4.94 (dd, *J* = 9.7 Hz, 2.3 Hz, 1H), 4.78 (d, *J* = 2.8 Hz, 1H), 4.39 (dd, *J* = 10.9 Hz, 6.7 Hz, 1H), 4.29 (d, *J* = 8.5 Hz, 1H), 4.20 (d, *J* = 8.5 Hz, 1H), 3.79 (d, *J* = 7.0 Hz, 1H), 2.60–2.47 (m, 1H), 2.38 (s, 3H), 2.36–2.31 (m, 1H), 2.31–2.25 (m, 1H), 2.23 (s, 3H), 1.91–1.84 (m, 1H), 1.79 (s, 3H), 1.68 (s, 3H), 1.23 (s, 3H), 1.14 (s, 3H). ^13^C NMR (126 MHz, CDCl_3_) δ 203.66, 172.77, 171.28, 170.42, 167.19, 167.03, 141.99, 138.04, 133.75, 133.70, 133.27, 132.02, 130.26, 129.25, 129.07, 128.77, 128.74, 128.40, 127.11, 127.09, 84.46, 81.22, 79.06, 76.56, 75.63, 75.03, 73.26, 72.41, 72.21, 58.66, 55.12, 45.71, 43.24, 35.76, 35.68, 26.91, 22.66, 21.87, 20.88, 14.86, 9.62. ESMS *m*/*z*: 876.3195 (M + Na)^+^.

### HPLC for Quantification and Purification

HPLC was utilized for both the quantification and purification of PTP and its degradation product, PTX, using a Shimadzu SPD-M20A system (Japan). Detection for both methods was carried out at 227 nm, and the column temperature was maintained at 25 °C. For quantification, a C_18_ column (4.6 × 150 mm, 5.0 μm, Shimadzu, Japan) was used. The mobile phase consisted of acetonitrile and 0.01 mol·L^–1^ disodium hydrogen phosphate (adjusted to pH 6.7 with phosphoric acid) with a constant 1:1 (v/v) ratio. The flow rate was 1.0 mL·min^–1^, and the sample injection volume was 5.0 μL. For purification, a larger C_18_ column (10 × 250 mm, 5.0 μm, Shimadzu, Japan) was employed. Fractions containing the purified compounds were collected using a fraction collector (FRC-10A, Shimadzu, Japan). A flow rate of 5.0 mL·min^–1^ and a sample injection volume of 50.0 μL were used for purification. The mobile phase for PTP purification consisted of acetonitrile and 0.01 mol·L^–1^ disodium hydrogen phosphate (pH adjusted to 6.7 with phosphoric acid) at a constant ratio of 57:43 (v/v). For PTX purification, the mobile phase consisted of acetonitrile and water at a constant ratio of 57:43 (v/v).

### Preparation of Formulations

PTP self-assemblies were prepared by adding 5.0 mg of PTP to 1.0 mL of a water, followed by vortexing for 1 min. For PTP@PEG coassemblies, 260.0 mg of PEG400 was mixed with 1.0 mL of the chosen water-based solution. This PEG400 solution was then added to 5.0 mg of PTP powder, and the resulting mixture was vortexed for 1 min. PTP@HA coassemblies were prepared by first dispersing 20.0 mg of HA in 1.0 mL of the selected water-based solution. Following this step, the resulting HA solution was added to 5.0 mg of PTP powder. The final mixture was vortexed for 1 min. To prepare the PTX@PEG control system, 1.3 g of PEG400 was mixed with 5.0 mL of water. Then, 25.0 mg of PTX was added to the solution, and the mixture was vortexed for 1 min. Similarly, the PTX@HA control system was prepared by dispersing 100.0 mg of HA in 5.0 mL of water. Subsequently, 25.0 mg of PTX was added, and the mixture was vortexed for 1 min.

### Surface Tension Measurement

Surface tension measurements were conducted at 25 °C using a drop shape analyzer (DSA30S; Kruss, Germany). Starting with a 5.0 mg·mL^–1^ PTP dispersion, serial dilutions were prepared to obtain a range of lower concentrations. Following overnight assembly, the PTP dispersion was drawn into a syringe (HENKE-JECT, Germany), and a droplet was suspended from the needle tip. The outer diameter of the needle (1.825 mm) served as the reference for image analysis. Images of the droplet were captured, and the surface tension was calculated using the Young–Laplace equation based on the droplet’s shape.

### Microscopy Imaging

For Cryo-TEM imaging, 5 μL of a 5 mg·mL^–1^ PTP system was applied onto Lacey Formvar/Carbon grids (200 mesh, Cu; Ted Pella Inc.) at room temperature within a controlled environmental chamber set at 97–99% humidity. Excess liquid was removed by blotting with filter paper for 2–3 s. The thin film was then rapidly vitrified by plunging it into liquid ethane cooled by liquid nitrogen. The grids were transferred to a Gatan 626 cryostat using a cryotransfer device and subsequently examined using a Talos F200C TEM (200 kV). Images were captured with an SC 1000 CCD camera (Gatan Inc., USA) at approximately −175 °C using an accelerating voltage of 200 kV.

For AFM imaging, a 5.0 mg·mL^–1^ dispersion of PTP or PTP@HA in water was prepared. After overnight assembly, 10 μL of the dispersion was placed on a mica sheet and allowed to dry completely under ambient conditions. Images were obtained using a Bruker Dimension Icon instrument with ScanAsyst-Air cantilever tips operating in ScanAsyst mode.

For polarizing microscopy imaging, an 8 μL dispersion of either PTP@HA (containing 5.0 mg·mL^–1^ PTP and 20 mg·mL^–1^ HA) or HA (20 mg·mL^–1^) was placed on a microscope slide and covered with a coverslip. Morphological analysis was conducted using a polarizing microscope (Axio Observer 3; Carl Zeiss, Germany) with a 5× objective. Images were captured using a Baumer camera (VLXN-06M.I; Frauenfeld, Switzerland).

### Cell Culture Conditions and Subculturing Procedure

The mouse breast cancer cell line 4T1 and the human ovarian cancer cell line SKOV3 were cultured in RPMI 1640 medium supplemented with 10% fetal bovine serum, 100 units·mL^–1^ penicillin, and 100 μg·mL^–1^ streptomycin. The human lung cancer cell line A549 and the luciferase-expressing human colorectal carcinoma cell line HCT116-Luc were cultured in high-glucose DMEM medium supplemented with 10% fetal bovine serum, 100 units·mL^–1^ penicillin, and 100 μg·mL^–1^ streptomycin. All cell lines were maintained at 37 °C in a Steri-Cycle 371 incubator (Thermo Scientific, USA) with a humidified atmosphere of 5% CO_2_. Only cells between passages 5 and 15 were used for cell-based experiments.

Cells were passaged when they reached approximately 90% confluence. The culture medium was removed, and the cells were rinsed twice with sterile PBS (pH 7.4). To detach the cells, 1 mL of a 0.25% trypsin-EDTA solution was added to the flask, which was then incubated for 2 min, followed by gentle tapping. The trypsin activity was neutralized by adding 2 mL of the corresponding complete growth medium. The resulting cell suspension was centrifuged at 1000 rpm for 3 min, and the supernatant was discarded. The cell pellet was then resuspended in 16 mL of complete growth medium, and 1 mL of this suspension was evenly distributed into a new flask. The culture medium was replaced every other day, and the cells were passaged approximately once a week.

### Cell Viability Assays

The antiproliferative activity of PTP was evaluated using the Cell Counting Kit-8 (CCK8) assay. PTX solution served as a positive control. Cell suspensions were prepared at a density of 3 × 10^4^ cells·mL^–1^ and seeded in 96-well plates at a volume of 100 μL per well. The plates were incubated for 24 h to allow cell attachment and growth. Subsequently, the culture media were replaced with fresh media containing varying concentrations of PTX or PTP solutions (100 μL per well), and the cells were incubated for an additional 48 h (*n* = 3). Control wells received fresh culture media alone (*n* = 3). Following a 48 h treatment, 10 μL of CCK8 reagent was added to each well. The plates were then shaken gently and incubated for 2 h. Finally, the absorbance was measured at 450 nm using a microplate reader (Synergy H1, BioTek, USA). The antiproliferative activity was quantified by calculating the 50% inhibitory concentration (IC_50_).

### Stability of PTP Assemblies in Solution, Plasma, and Solid State

PTP, PTP@PEG, and PTP@HA (200 μL, 0.1 mg·mL^–1^) solutions were sealed in ampules and incubated at 37 °C with agitation at 100 rpm. Samples were collected in triplicate at scheduled intervals, treated with acetonitrile, vortexed for 1 min, and analyzed using HPLC. Degradation rate constants (*k*) were calculated by plotting the natural logarithm of the concentration ratio (ln c/c_0_) versus time, with the slopes of the linear regression plots representing the rate constants.

For plasma stability studies, a PTP or PTP@PEG dispersion (1.2 mg·mL^–1^, PTP-equivalent) was diluted with plasma to a final concentration of 100.0 μg·mL^–1^. Plasma was obtained from male rat blood by centrifugation (1600 × *g*, 15 min). Aliquots (100 μL each) were incubated at 37 °C with agitation at 100 rpm, and samples were collected at designated time points in triplicate. Proteins were precipitated by adding 200 μL acetonitrile, followed by vortex mixing and centrifugation at 17 000 rpm and 4 °C for 30 min. The resulting supernatants were analyzed by HPLC.

For solid-state stability, 5.0 mg PTP was sealed in glass bottles and stored under controlled conditions in stability chambers (MMM Climacell-E, Germany). The samples were stored at 40 ± 2 °C and 75 ± 5% relative humidity, and 25 ± 2 °C and 60 ± 5% RH. Additional samples were stored at 4 °C and −20 °C in a refrigerator (BCD-610W, Siemens, Germany). At scheduled intervals, triplicate samples from each condition were analyzed for purity by HPLC.

### Isothermal Titration Calorimetry Analysis

ITC experiments were performed using a Nano-ITC (TA Instruments, USA) with all samples degassed for 8 min prior to measurement. Experiments were conducted under stirring at 350 rpm. To account for background signals, control experiments were conducted and subtracted from the test data. The resulting heat values were analyzed using an independent model to extract thermodynamic parameters. For tubulin binding studies, solutions were prepared in PEM buffer containing 5% (v/v) DMSO. In each experiment, 400 μL of the titrant was loaded into the sample cell, and 400 μL of PEM buffer with 5% (v/v) DMSO was placed in the reference cell. Tubulin solution (180 μM) was titrated into the sample cell in 25 injections of 2 μL each at 4 °C. A control titration of tubulin into PEM buffer (5% DMSO) was performed to account for dilution effects. To assess interactions between PTP and excipients, PTP dispersions (25 μM) were loaded into the sample cell, and water was used in the reference cell. PEG400 solution (6.0 mg·mL^–1^) was titrated into a 2.0 mg·mL^–1^ PTP dispersion, and HA (2.7 mg·mL^–1^) into a 1.0 mg·mL^–1^ PTP dispersion. Both titrations were performed at 25 °C with 25 injections of 2 μL each. Control experiments were performed by titrating PEG400 or HA into water to correct for nonspecific heat effects.

### *In Vitro* PTP Release

The *in vitro* drug release from the PTP assemblies and PTP@HA was assessed in 1 × PBS (pH 7.4). A 0.1 mL dispersion of PTP (5.0 mg·mL^–1^) or PTP@HA (equivalent to 5.0 mg·mL^–1^ PTP and 20.0 mg·mL^–1^ HA) in saline was added to the upper chamber of a 24-well transwell plate (#3413, Corning, USA) equipped with a 0.4 μm polycarbonate membrane (*n* = 3). The lower chamber contained 0.6 mL 1 × PBS. The setup was maintained at 37 °C with shaking at 100 rpm. At specified time intervals, the PBS in the lower chamber was replaced with fresh PBS. Each collected sample was mixed with 600 μL acetonitrile and vortexed for 1 min. PTP release and PTX degradation products were quantified by HPLC.

### Animal Studies

All animal experiments were approved by the China Pharmaceutical University Institutional Animal Care and Use Committee (Approval No. 202102006) and performed according to institutional guidelines. Animals were acclimatized for 1 week prior to experiments with free access to food and water.

### Sample Preparation for LC-MS Analysis

For plasma samples, 50 μL of plasma was mixed with 100 μL of a docetaxel acetonitrile solution (15 ng·mL^–1^) for protein precipitation. The mixture was vortexed and then centrifuged at 4 °C, 17 000 rpm for 30 min. For tissue samples, mice were euthanized at specified time points, and tissues were harvested and rinsed with saline. Two kidneys, the entire heart, spleen, lung, tumor, and 150–200 mg of liver were placed in grinding tubes. Saline was added at a ratio of 1:5 (m/v), and the tissues were ground using a freezing grinder (Jingxin, China) at 4 °C. Next, 100 μL of docetaxel acetonitrile solution (15 ng·mL^–1^) was added to 50 μL of the tissue homogenate. After vortex mixing, the mixture was centrifuged at 4 °C, 17 000 rpm for 30 min. The supernatants for both plasma and tissue samples were analyzed by LC-MS as described below.

### LC-MS Analysis

The concentrations of PTP and PTX in plasma and tissue samples were determined using liquid chromatography–mass spectrometry (LC-MS) with a G6470A system (Agilent, USA) equipped with a C_18_ column (2.1 × 100 mm, 2.7 μm, Agilent, USA). The mobile phase consisted of methanol (mobile phase A) and a solution of 10 mM ammonium acetate containing 5% (v/v) methanol (mobile phase B), maintained at a constant ratio of 62:38 (v/v) with a flow rate of 0.4 mL·min^–1^. The sample injection volume was 4.0 μL, and the column temperature was maintained at 25 °C. Mass spectrometry analysis for PTP, PTX, and the internal standard docetaxel were performed in positive ion mode using multiple reaction monitoring. The gas temperature and flow were set to 300 °C and 10 L·min^–1^, respectively. While the sheath gas temperature and flow were maintained at 350 °C and 11 L·min^–1^, with a nebulizer pressure of 45 psi. The capillary voltage was set to 3500 V, and the nozzle voltage at 500 V. The specific compound parameters were: PTP, fragment voltage 180 V, collision energy 30 eV, ion transition *m*/*z* 1012.2 → 444.1; PTX, fragment voltage 130 V, collision energy 28 eV, ion transition *m*/*z* 876.5 → 308.1; and docetaxel, fragment voltage 160 V, collision energy 26 eV, ion transition *m*/*z* 830.4 → 549.3.

### Pharmacokinetics, Urinary Excretion, and Biodistribution of PTP@PEG

Pharmacokinetic features were evaluated in Sprague–Dawley rats (200–300 g, *n* = 6 per group) following a single intravenous administration of PTX solution (1.0 mg·mL^–1^), PTP (1.2 mg·mL^–1^), or PTP@PEG (1.2 mg·mL^–1^) at a dose equivalent to 3.0 mg·kg^–1^ PTX. Blood samples were collected at scheduled time intervals, and plasma samples were obtained by centrifugation (1600 × *g*, 15 min). For urinary excretion analysis, Sprague–Dawley rats (*n* = 6 per group) received the formulations intravenously at 3.0 mg·kg^–1^ PTX equivalent (corresponding to 3.0 μL·g^–1^ injection volume). Urine was collected using metabolic cages at scheduled time points, centrifuged (1600 × *g*, 15 min), and the supernatant retained. Biodistribution was assessed in female Balb/c mice (18–22 g; *n* = 6 per group) bearing subcutaneous 4T1 tumors (target volume 200–300 mm^3^). Mice received a single intravenous dose of PTX solution, PTP, or PTP@PEG at 10.0 mg·kg^–1^ PTX equivalent. Mice were euthanized at 1, 4, and 8 h postdosing, and heart, liver, spleen, lung, kidney, and tumor tissues were harvested. Drug concentrations in plasma, urine supernatant, and tissue homogenates were quantified using LC-MS (G6470A, Agilent, USA). Pharmacokinetic parameters were calculated from the plasma drug concentration–time data using PKsolver.^84^

### Pharmacodynamics and Safety Assessment of PTP@PEG

For pharmacodynamic evaluation, female Balb/c mice (18–22 g, *n* = 6 per group) bearing subcutaneous 4T1 tumors (approximately 50 mm^3^) received intravenous injections every other day for five doses at 10.0 mg·kg^–1^ (PTX equivalent). Tumor volume and body weight were monitored daily until day 14, when tumors were excised and photographed. At study termination (day 14), major organs (heart, liver, spleen, lungs, and kidneys) were collected from these mice. The organs were fixed in 4% paraformaldehyde, dehydrated with ethanol, and cleared with xylene. Afterward, the tissues were embedded in paraffin wax and sectioned after dipping wax operation at 60 °C. The sections were then stained with hematoxylin and eosin for histological examination.

In a related safety assessment, female Balb/c mice (18–22 g, *n* = 3) were fasted for 12 h, then treated intravenously with either 5% G, PTX solution, PTP, or PTP@PEG using the previously described dosing regimen (10 mg·kg^–1^ PTX equivalent, q.o.d. × 5). On day 10, blood was collected and then processed to obtain serum by centrifugation (2000 rpm, 10 min, 4 °C). Serum samples were subsequently analyzed for biochemical markers using a Chemray 240 automated analyzer (Rayto Life and Analytical Sciences Co., Ltd., China).

The maximum tolerated dose of PTP was determined in female Balb/c mice (18–22 g, *n* = 6 per group) receiving four intravenous injections (4 h apart) of 5% glucose (control), PTX (total dose of 30 mg·kg^–1^), or PTP (total dose of 100 mg·kg^–1^). These mice were monitored for body weight and clinical signs for 14 days.

### Pharmacokinetics of PTP@HA

The PTP@HA hydrogel, containing PTP at a concentration of 5.0 mg·mL^–1^, was prepared by adding HA saline solution (20.0 mg·mL^–1^) to solid PTP, followed by vortexed for 1 min. Male Sprague–Dawley rats (200–300 g, *n* = 6 per group) received a single intraperitoneal injection (20.0 μL·g^–1^) of either PTX solution (delivering 12.0 mg·kg^–1^ PTX) or PTP@HA hydrogel (delivering 100.0 mg·kg^–1^ PTP). Following administration, blood samples were collected from the rats at scheduled time-points appropriate for the expected pharmacokinetic profile of the administered formulation. All collected blood samples were centrifuged (1600 × *g*, 15 min) to obtain plasma. Plasma concentrations of both PTP and PTX were subsequently quantified using LC-MS (G6470A, Agilent, USA).

### Pharmacodynamics of PTP@HA

An intraperitoneal tumor model was established by injecting male Balb/c nude mice (18–22 g) with 100 μL of HCT116-Luc cells (10^7^ cells·mL^–1^). One week later, baseline tumor burden was imaged using a Tanon ABLX6 system following intraperitoneal administration of 150.0 mg·kg^–1^ D-Luciferin potassium salt (15.0 mg·mL^–1^; 10.0 μL·g^–1^). Following baseline imaging, mice were randomly assigned (*n* = 6 per group) to receive treatment starting on day one. Treatments were administered intraperitoneally as follows: the control group received a single injection of saline (9.0 μL·g^–1^); the PTX solution group received three injections (15.0 mg·kg^–1^ PTX per injection; 25.0 μL·g^–1^ each) at 4 h intervals, totaling 45.0 mg·kg^–1^ PTX; and the PTP@HA group received a single injection of the hydrogel delivering 45.0 mg·kg^–1^ PTX equivalent (9.0 μL·g^–1^), matching the total PTX dose. Tumor progression was subsequently monitored by weekly *in vivo* imaging for 2 weeks, and animal body weight was recorded daily. At the end of the study, mice were euthanized and dissected. Antitumor efficacy was evaluated using the ePCI scoring system and by photographing the peritoneal tumors.

### Statistical Analysis

Results are expressed as the mean ± SD for at least three independent experiments. Significance of difference was analyzed using one-way analysis of variance (ANOVA) followed by a Bonferroni’s posthoc test for multiple comparisons; the levels of significance were set at ***p < 0.001; **p < 0.01; *p < 0.05; *N.S.*, not statistically significant.

## Supplementary Material



## Notes

The synthetic route and analytical data for PTP and its intermediates (Scheme S1, Figures S1–S8 and S31); PTP’s properties, including morphology, molecular interactions, degradation, and stability analysis (Figures S9–S14); molecular dynamics simulations for excipient screening (Figures S15–S17 and S20–S22); analysis of PTP interactions with PEG400 or HA, including visual characterization (Figures S18, S23, and S24) and degradation studies (Figures S19 and S25); and *in vivo* efficacy and safety profiles for PTP@PEG (Figures S26–S29), along with a comparative study of PTP versus PTX solution (Figure S30) (PDF)
