# Supplementary material for: Programmable Nanostructure Assembly of a Paclitaxel Derivative Enables Tunable Anticancer Therapy via Hydrogen Bond Engineering
Source: ACS Nano. 2025 Aug 26;19(35):31799–817. doi: 10.1021/acsnano.5c10267 (PMC12424289; doi:10.1021/acsnano.5c10267)
Supplement: Supplementary file 1 [file nn5c10267_si_001.pdf]

Programmable Nanostructure Assembly of a Paclitaxel Derivative Enables Tunable Anticancer Therapy via Hydrogen Bond Engineering

Guobing Feng[1,2], Hui Tang[1,3], Shuyi Xie[1], Yingying Wang[1], Tongyu Wu[1], Xiongru Cai[1], Yunyi Zhou[1], Yan Lu[1], Yuancheng Bai[1], Mengfan Zhao[1], Shuai Hu[1,4], Yuezhou Zhang[5], Mohammad-Ali Shahbazi[*4], Hélder A. Santos[*4], Jin Fan[*3], Dongfei Liu[*1]

[1]State Key Laboratory of Natural Medicines, School of Pharmacy, China Pharmaceutical University, Nanjing 210009, China, E-mail: dongfei.liu@cpu.edu.cn

[2]Hangzhou Geriatric Hospital, Department of Pharmacy, Affiliated Hangzhou First People's Hospital Chengbei Campus, School of Medicine, Westlake University, Hangzhou 310022, China

[3]Department of Orthopaedics, The First Affiliated Hospital of Nanjing Medical University, Nanjing 210029, China, E-mail: fanjin@njmu.edu.cn

[4]Department of Biomaterials and Biomedical Technology, The Personalized Medicine Research Institute (PRECISION), University Medical Center Groningen, University of Groningen, Ant. Deusinglaan 1, Groningen 9713 AV, The Netherlands, E-mail: h.a.santos@umcg.nl; m.a.shahbazi@umcg.nl

[5]Frontiers Science Center for Flexible Electronics, Shaanxi Institute of Flexible Electronics, Northwestern Polytechnical University, Xi’an 710072, China

Keywords: *hydrogen bonds, assembly morphology, antitumor agents, renal accumulation, self-assembly*

Supplementary data

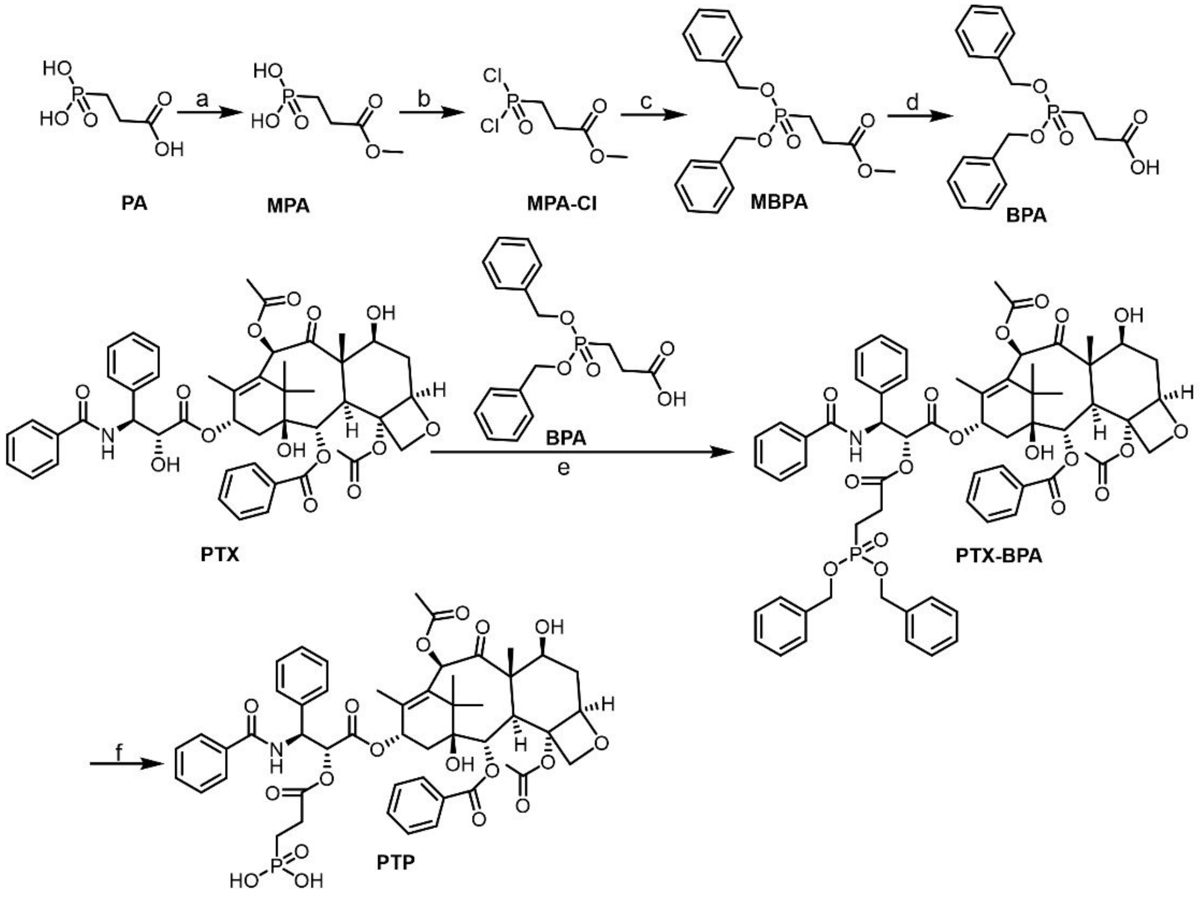

Scheme S1. The synthesis route of PTP. (a) room temperature, 4 days; (b) sulfinyl chloride, DMF, 60 °C, 1h; (c) benzyl alcohol, DCM, pyridine, DIPEA, ice bath→room temperature, overnight; (d) THF, NaOH, room temperature, 2.5 h; (e) DCM, EDCI, DMAP, HOBt, ice bath 3 h; PTX, room temperature 6 h; (f) methanol, $HCOONH_4$, 10% Pd/C, 50 °C, 30 min.

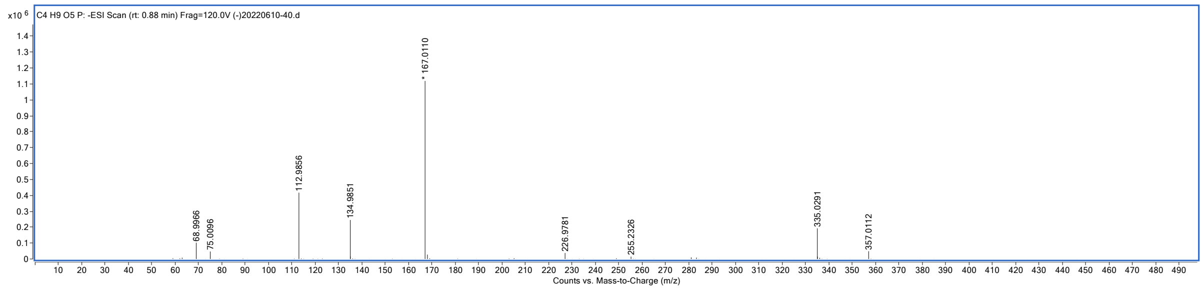

Fig. S1. Mass spectrum of MBA.

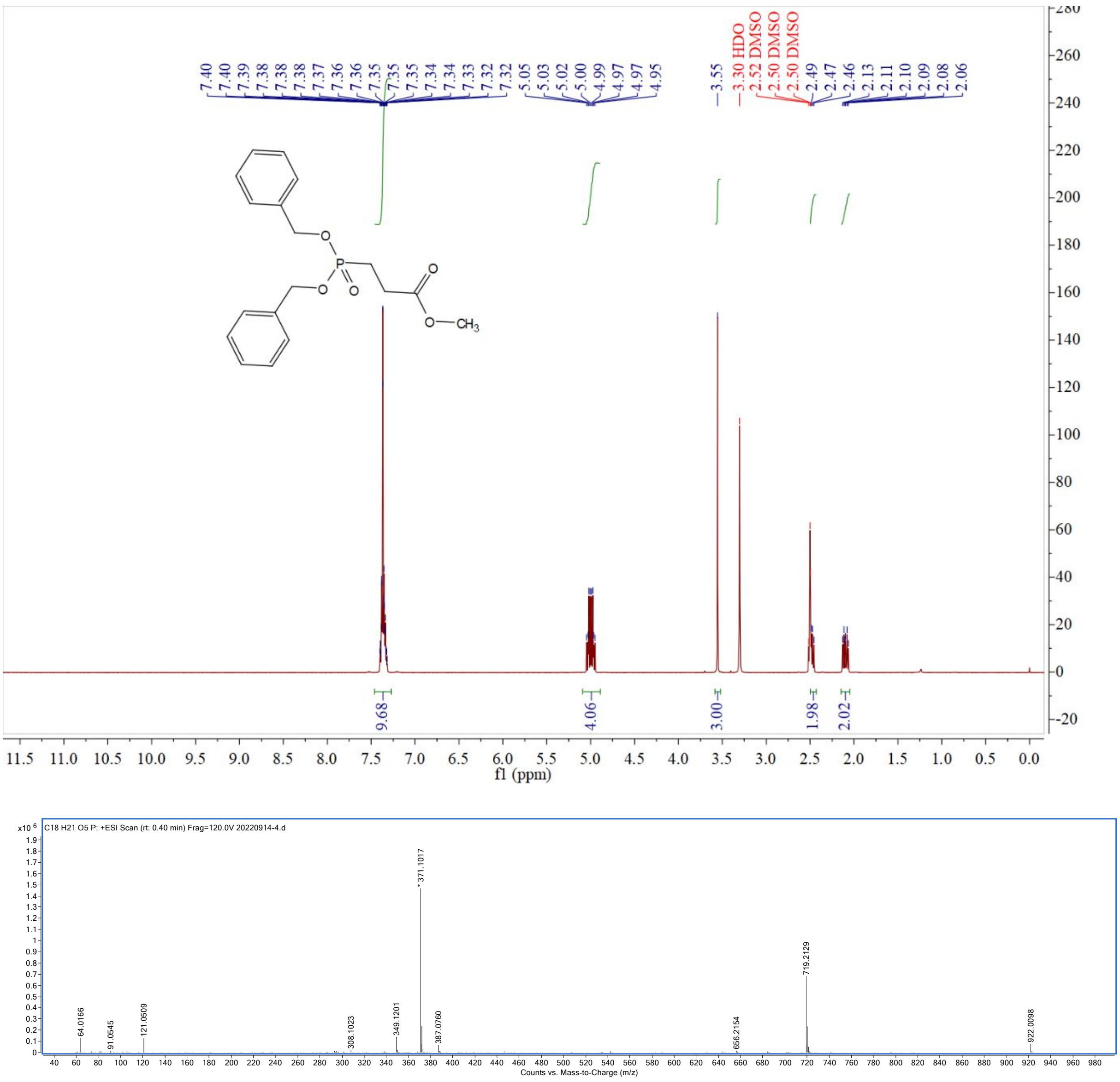

Fig. S2. The ^{1}H NMR and mass spectra of MBPA.

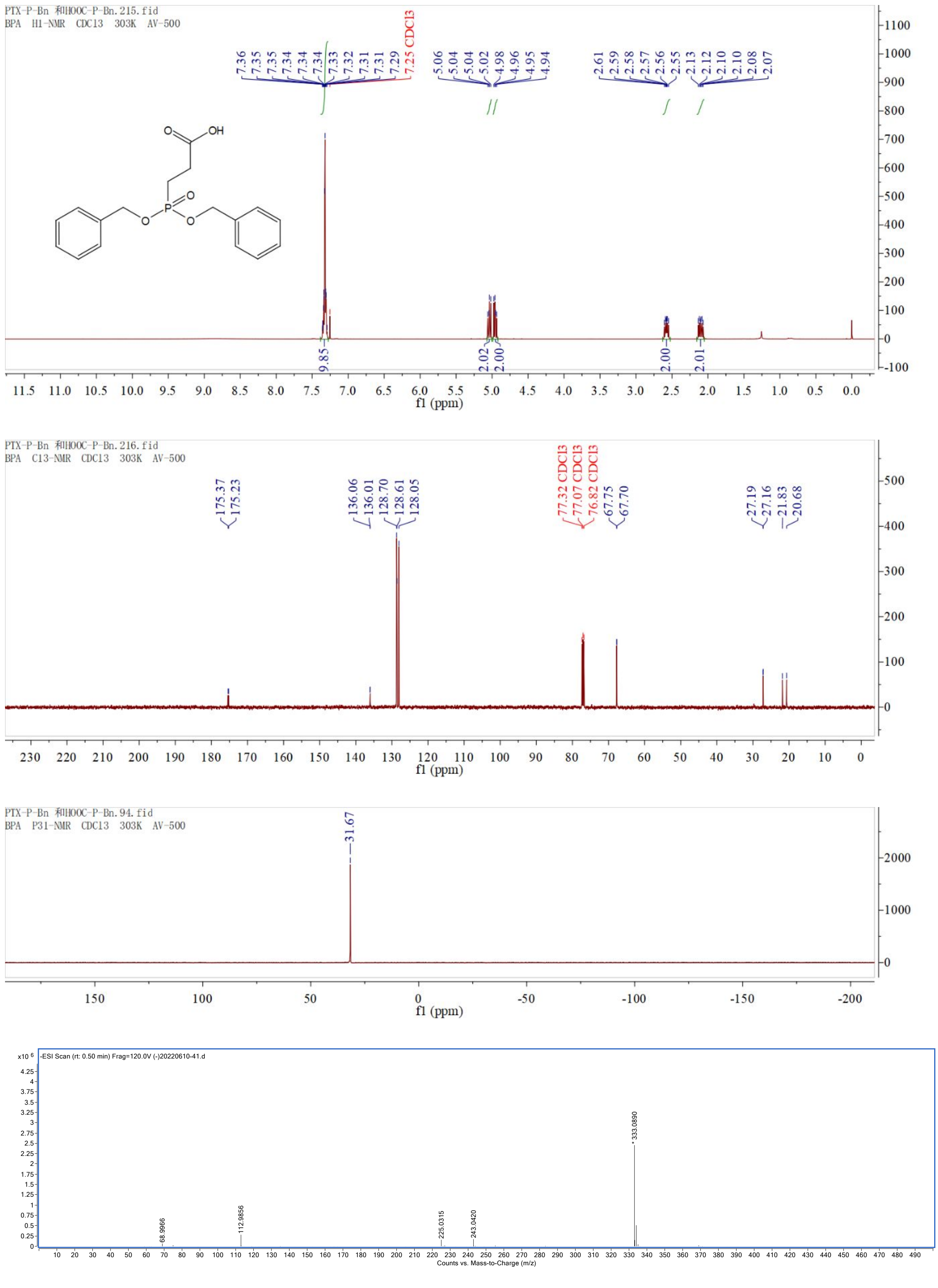

Fig. S3. The ^{1}H NMR, ^{13}C NMR, ^{31}P NMR and mass spectra of BPA.

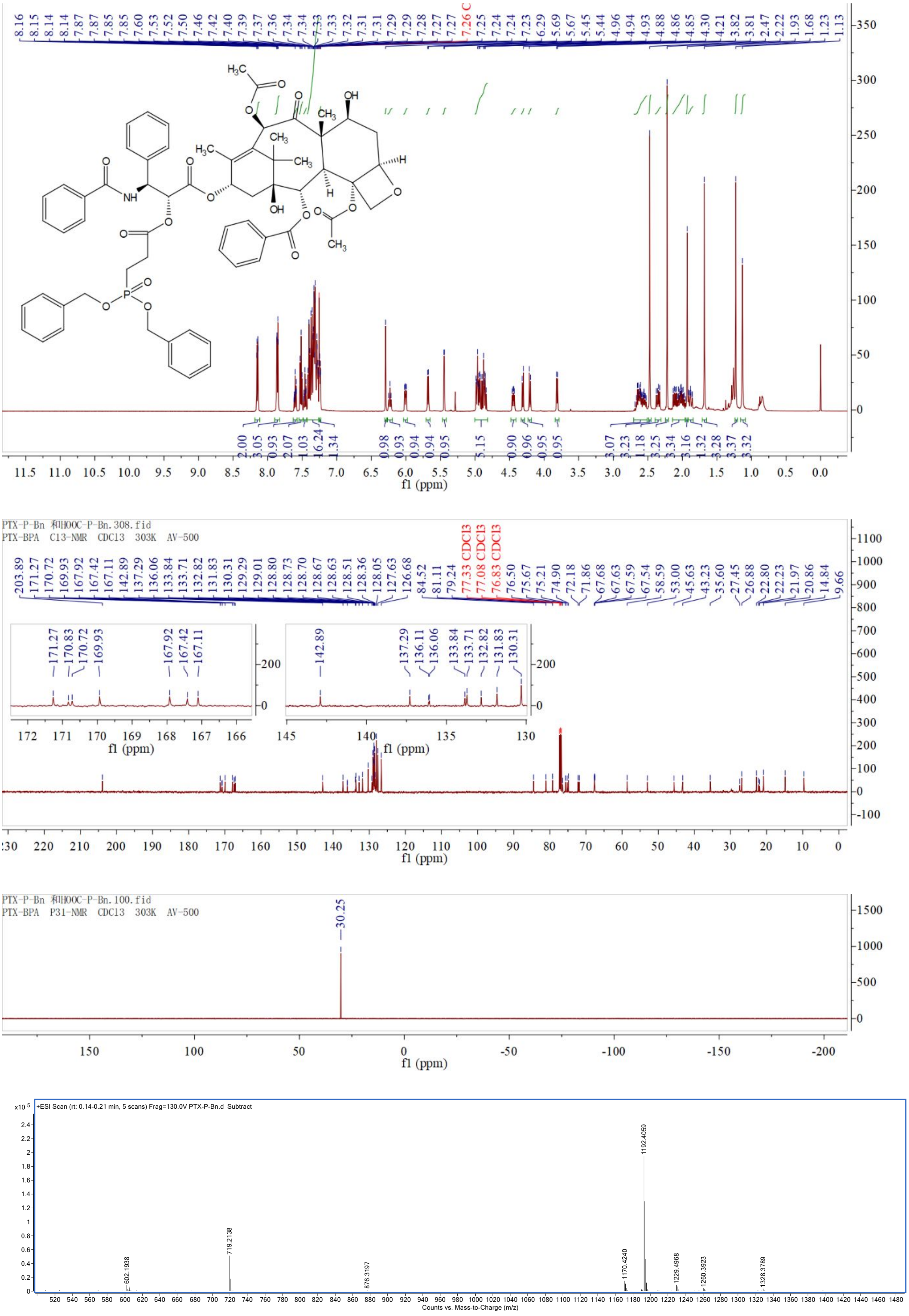

Fig. S4. The ^{1}H NMR, ^{13}C NMR, ^{31}P NMR and mass spectra of PTX-BPA.

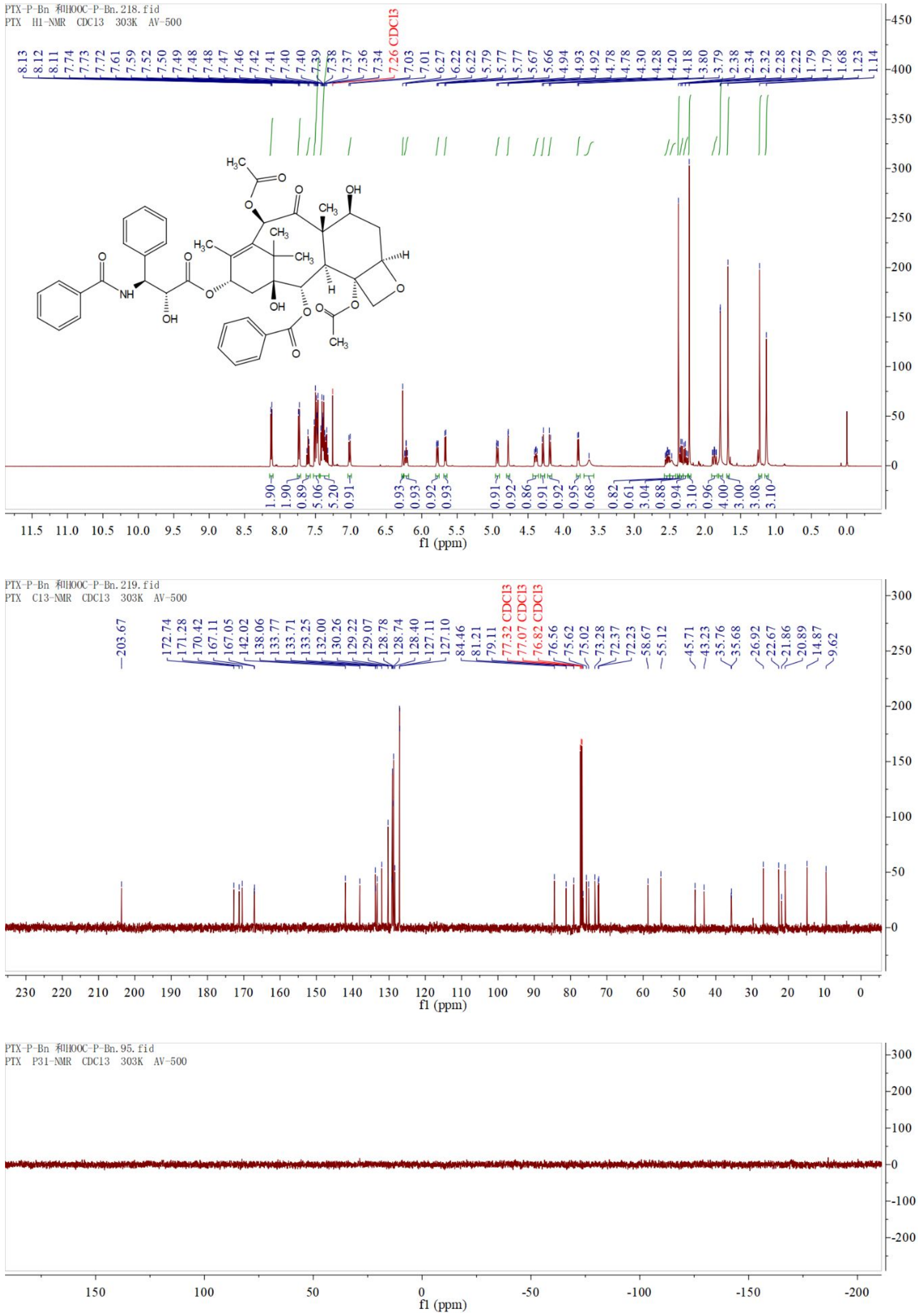

Fig. S5. The ^{1}H NMR, ^{13}C NMR and ^{31}P NMR spectra of PTX.

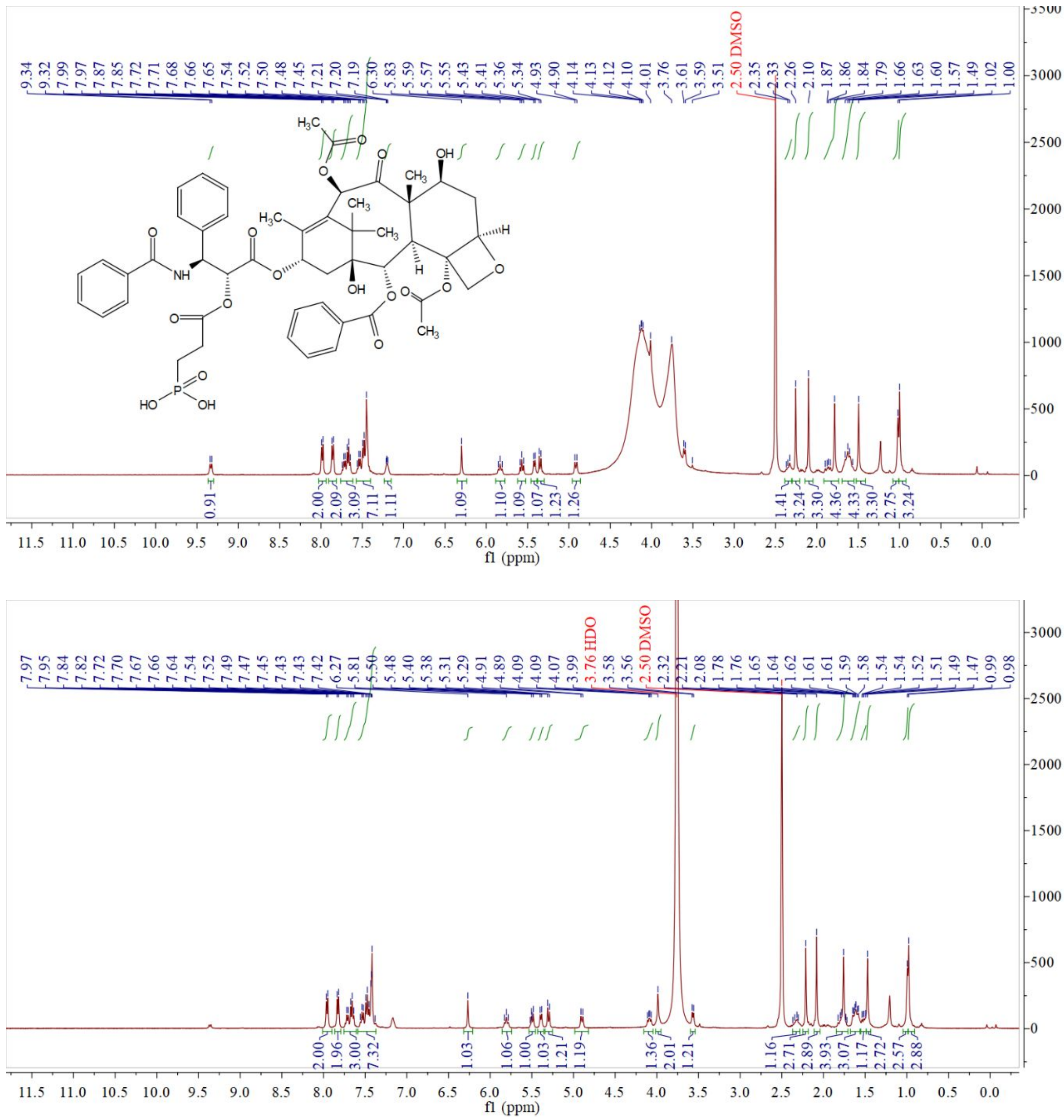

Fig. S6. ^{1}H NMR spectra of PTP recorded in DMSO-d_6 (upper panel) and following the addition of D_2O (lower panel).

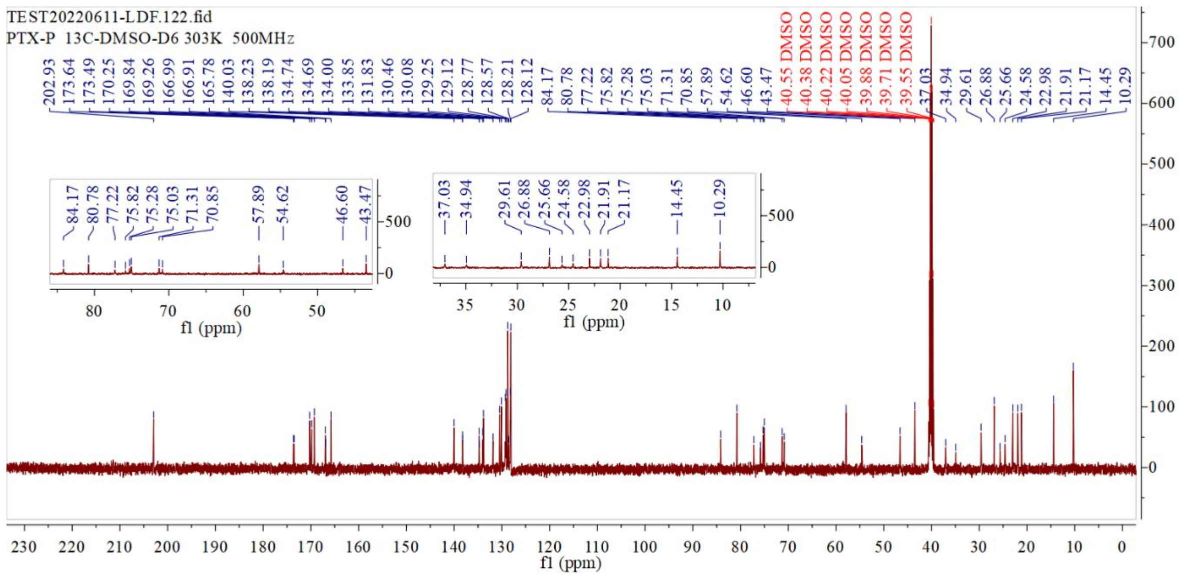

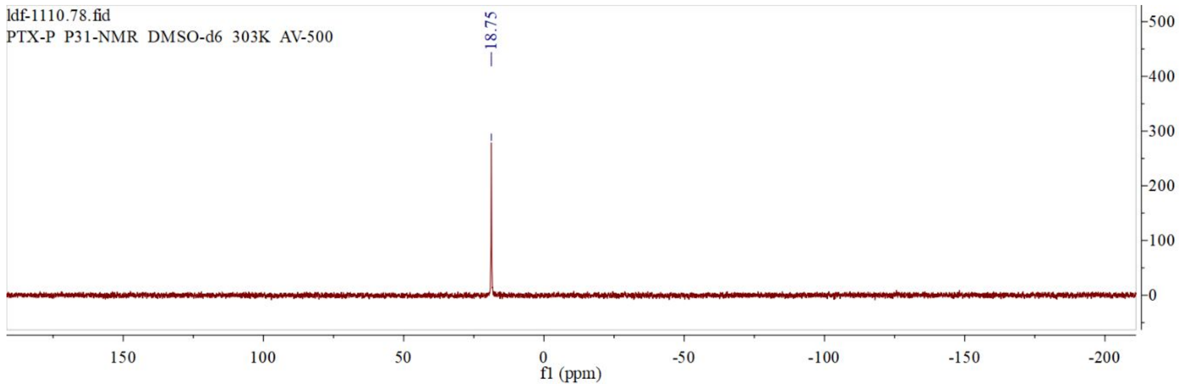

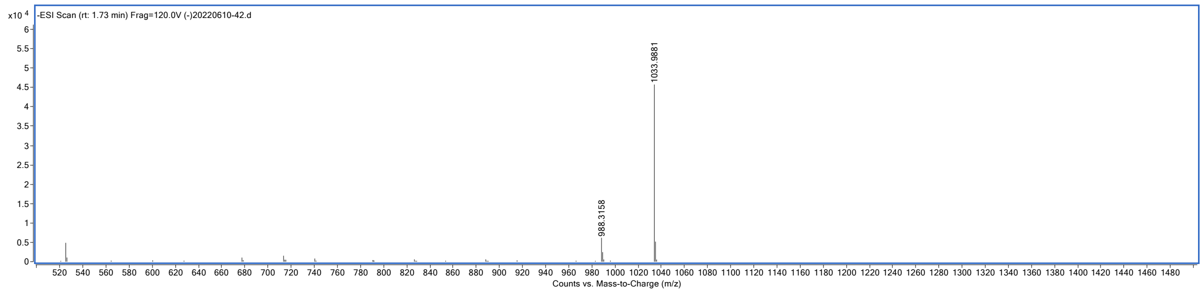

Fig. S7. The ^{13}C NMR, ^{31}P NMR and mass spectra of PTP.

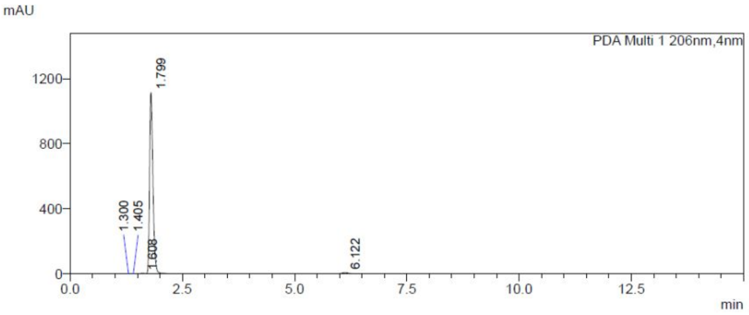

PDA Ch1 206nm

Peak#	Ret. Time	Area	面积%	分离度(USP)
1	1.300	10963	0.183	--
2	1.405	8186	0.137	0.473
3	1.608	23360	0.390	0.952
4	1.799	5871992	98.032	1.164
5	6.122	75384	1.259	20.069
总计		5989885	100.000	

Fig S8. HPLC chromatogram of PTP, recorded at 206 nm. Retention times and peak areas are indicated in the inset table.

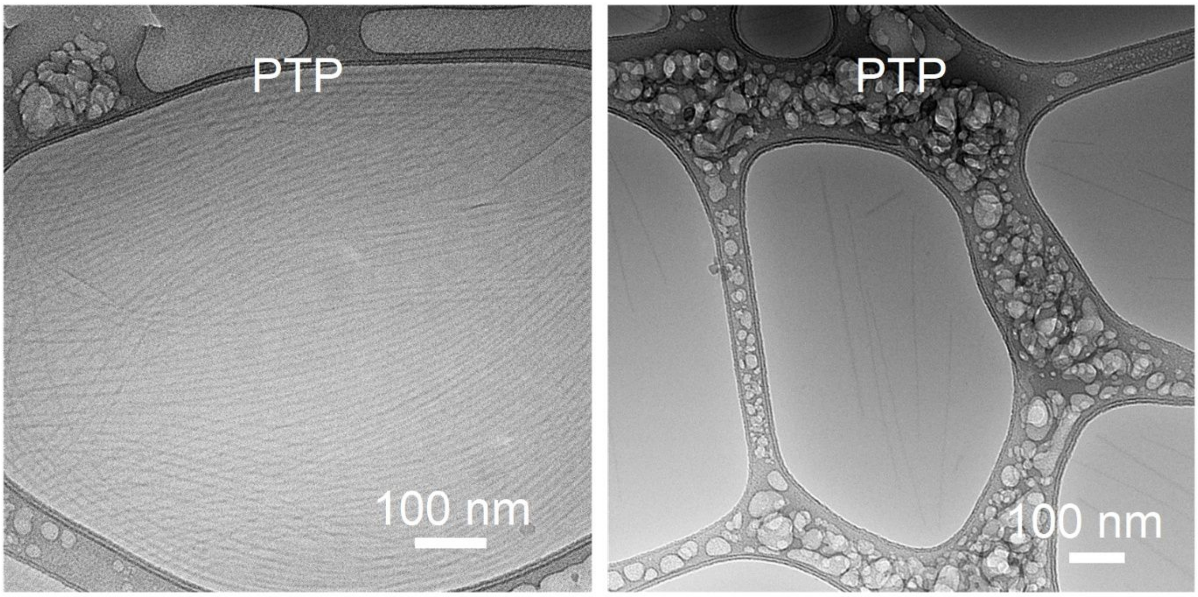

Fig. S9. Cryo-TEM images of the PTP system (5.0 mg·mL^{-1}).

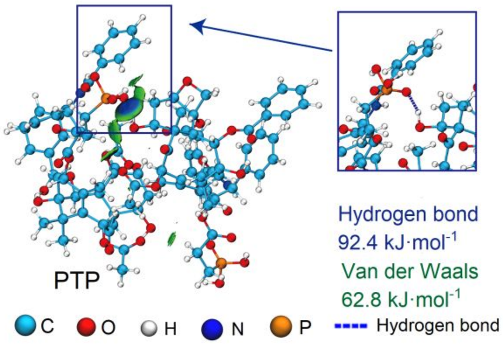

Fig. S10. Interaction energy calculations showing the relative strengths of hydrogen bonding and van der Waals interactions between PA and PTX' fragments.

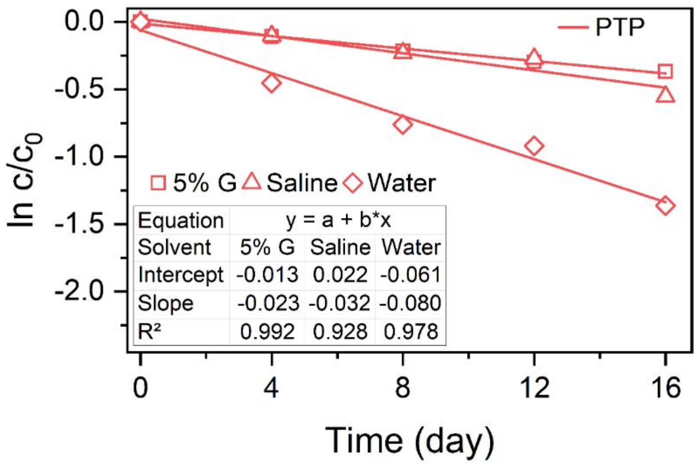

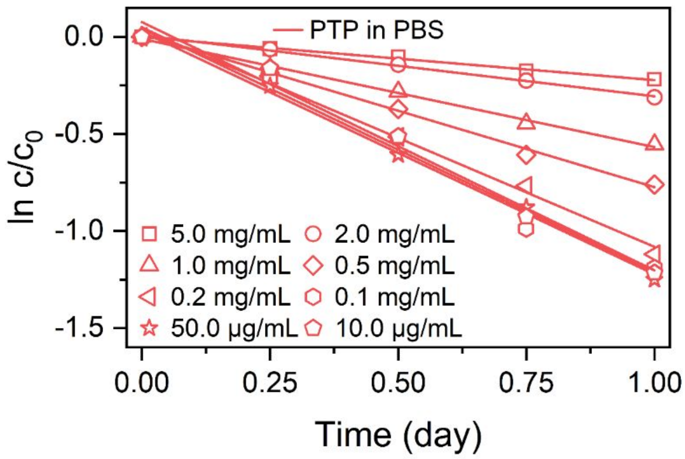

Equation	y = a + b*x							
Concentration	5.0 mg/mL	2.0 mg/mL	1.0 mg/mL	0.5 mg/mL	0.2 mg/mL	0.1 mg/mL	50.0 μg/mL	10.0 μg/mL
Intercept	-0.001	0.008	-0.010	0.013	0.040	0.050	0.027	0.076
Slope	-0.221	-0.314	-0.557	-0.787	-1.122	-1.261	-1.250	-1.279
R^2	0.994	0.997	0.996	0.995	0.993	0.982	0.996	0.983

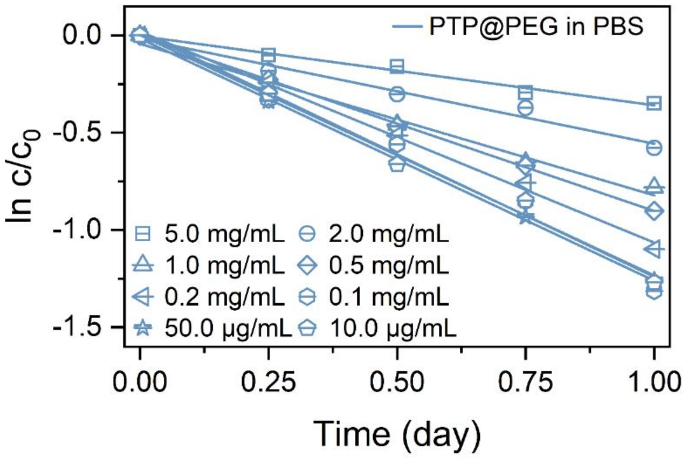

Equation	y = a + b*x							
Concentration	5.0 mg/mL	2.0 mg/mL	1.0 mg/m	0.5 mg/mL	0.2 mg/mL	0.1 mg/mL	50.0 μg/mL	10.0 μg/mL
Intercept	-0.003	-0.017	-0.043	-0.004	0.020	0.019	-0.016	0.002
Slope	-0.356	-0.539	-0.779	-0.898	-1.084	-1.259	-1.245	-1.236
R^2	0.984	0.977	0.985	0.999	0.996	0.985	0.999	0.990

Fig. S11. The degradation rate of PTP in PTP and PTP@PEG systems.

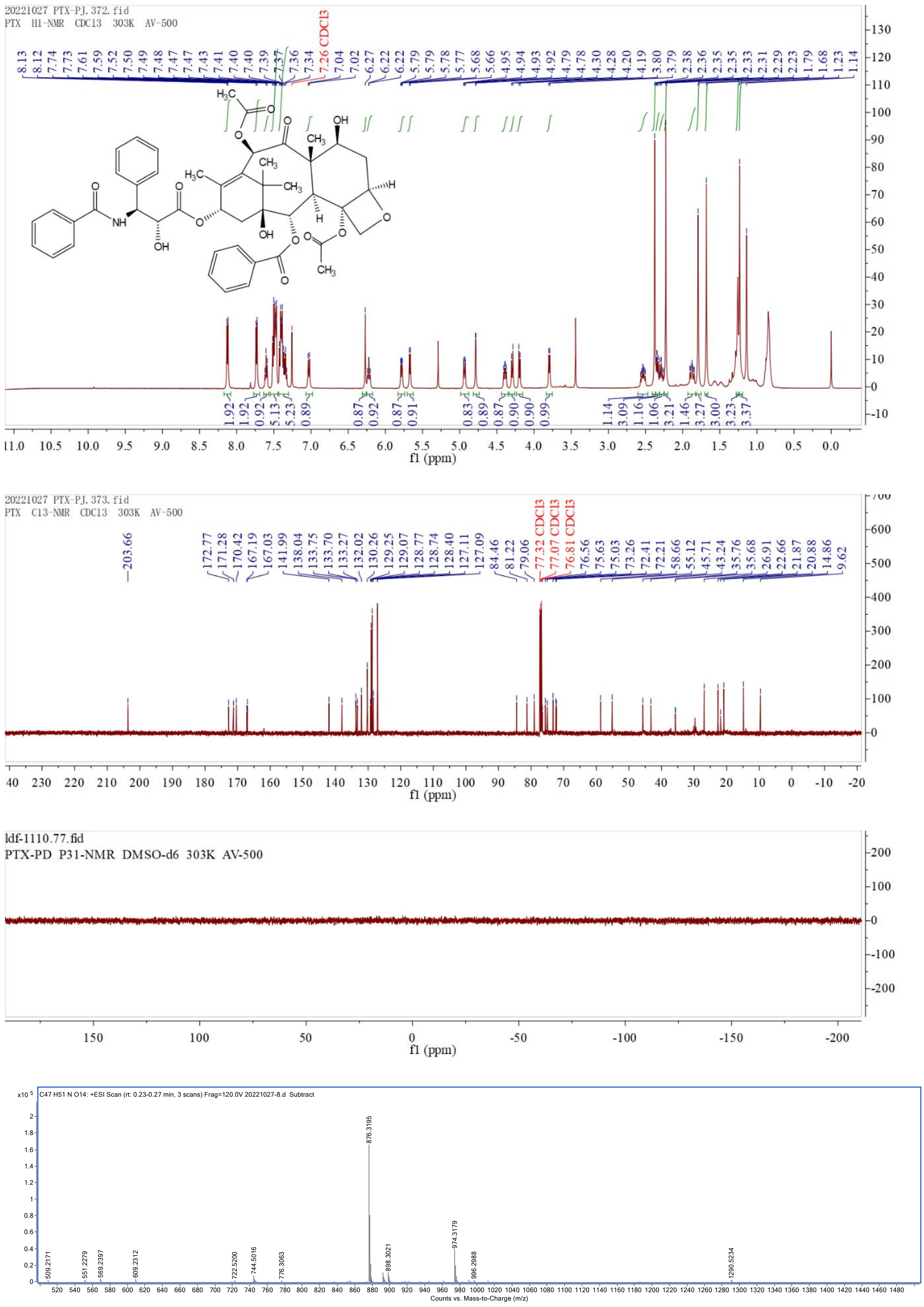

Fig. S12. The ^{1}H NMR, ^{13}C NMR, ^{31}P NMR and mass spectra of the major degradation product of PTP.

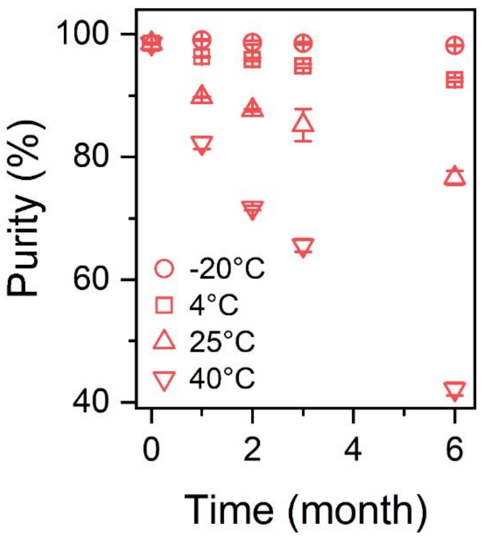

Fig. S13. Temperature-dependent stability of PTP.

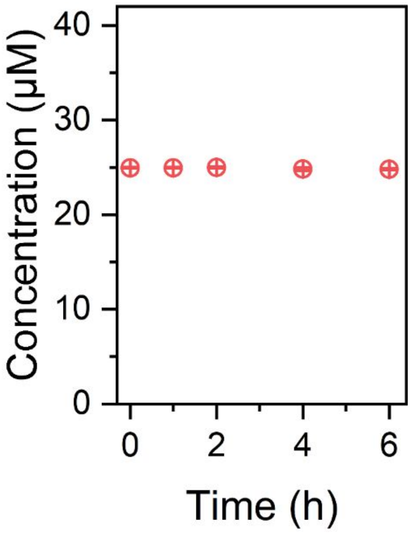

Fig. S14. Stability of PTP in PEM buffer containing 5% DMSO (v/v).

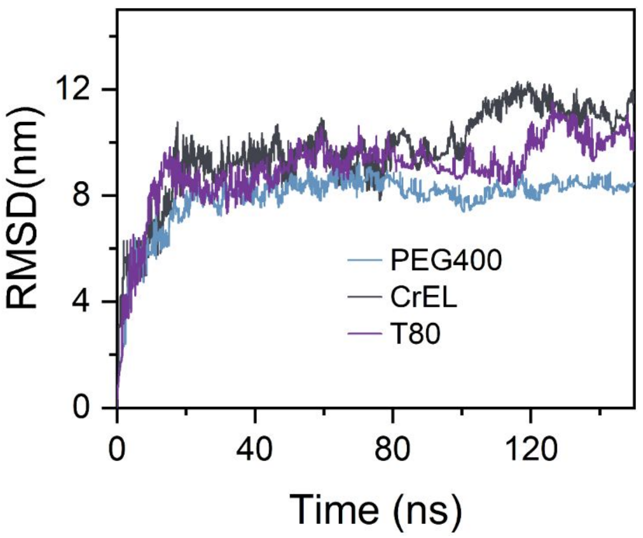

Fig. S15. Structural deviation of PTP during molecular dynamics simulations with common pharmaceutical excipients. RMSD from the initial structure for PTP is plotted against simulation time in systems containing PEG400, CrEL, or T80.

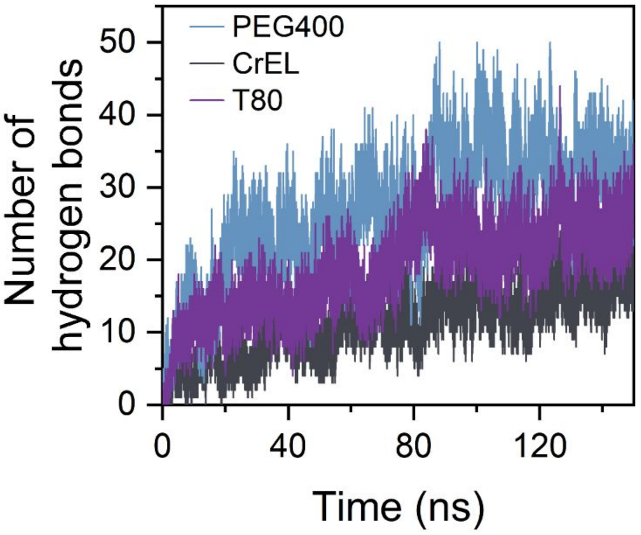

Fig. S16. Hydrogen bond analysis from molecular dynamics simulations. The plot tracks the number of hydrogen bonds formed between PTP and excipients (PEG400, CrEL or T80) throughout the simulation trajectory.

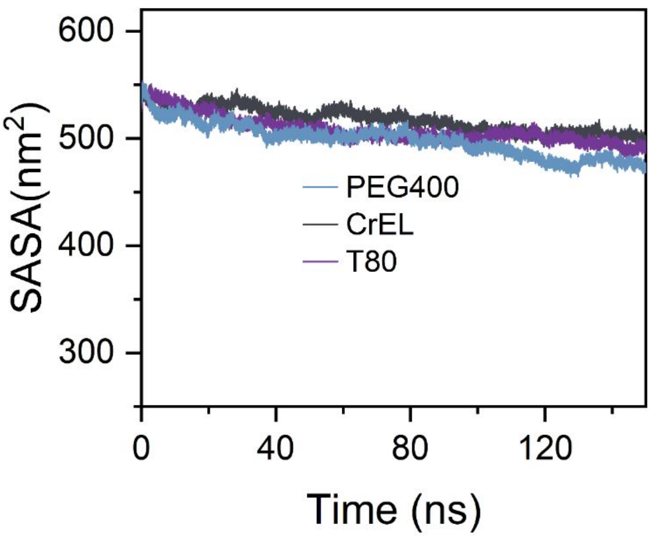

Fig. S17. Assessment of solvent exposure *via* SASA from molecular dynamics simulations. The plot shows the SASA of PTP over the simulation trajectory when interacting with excipients (PEG400, CrEL, or T80).

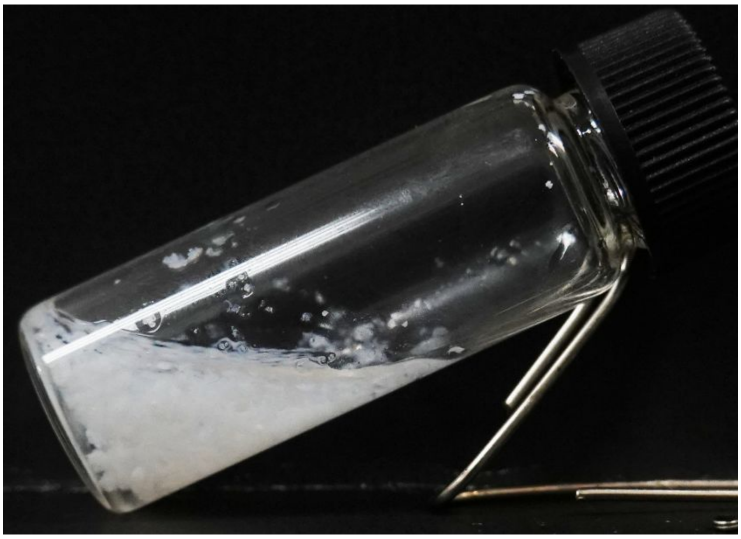

Fig. S18. Photograph of the PTX@PEG (prepared with 5.0 mg·mL^{-1} PTX and 260.0 mg·mL^{-1} PEG400).

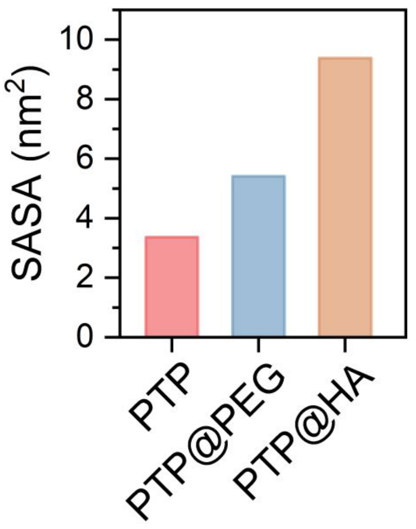

Fig. S19. The SASA of ester bonds in PTP alone, and in combination with PEG400 or HA.

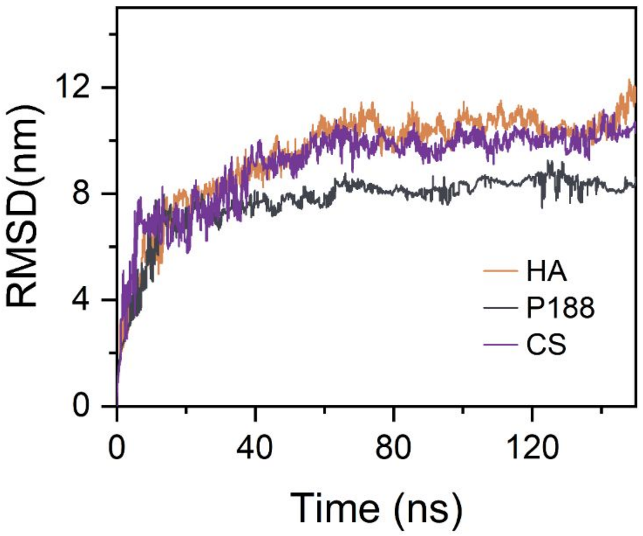

Fig. S20. Structural deviation of PTP during molecular dynamics simulations with common pharmaceutical excipients. RMSD from the initial structure for PTP is plotted against simulation time in systems containing HA, P188, or CS.

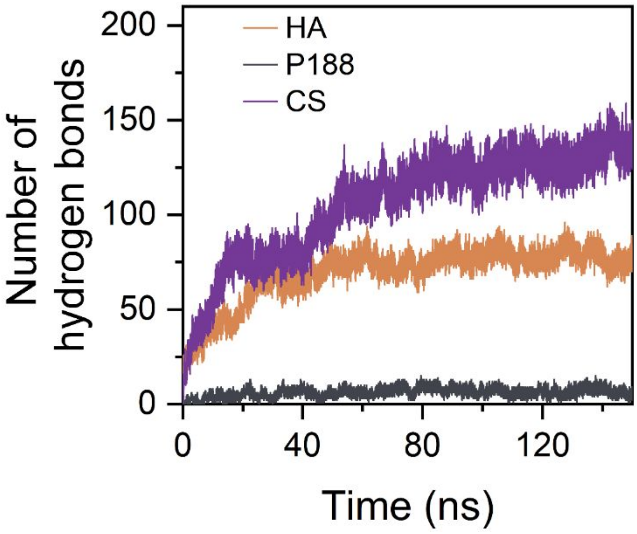

Fig. S21. Hydrogen bond analysis from molecular dynamics simulations. The plot tracks the number of hydrogen bonds formed between PTP and excipients (HA, P188, or CS) throughout the simulation trajectory.

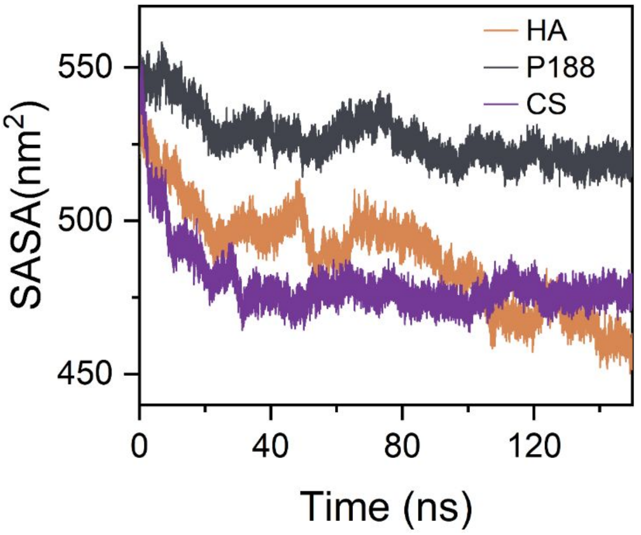

Fig. S22. Assessment of solvent exposure *via* SASA from molecular dynamics simulations. The plot shows the SASA of PTP over the simulation trajectory when interacting with excipients (HA, P188, or CS).

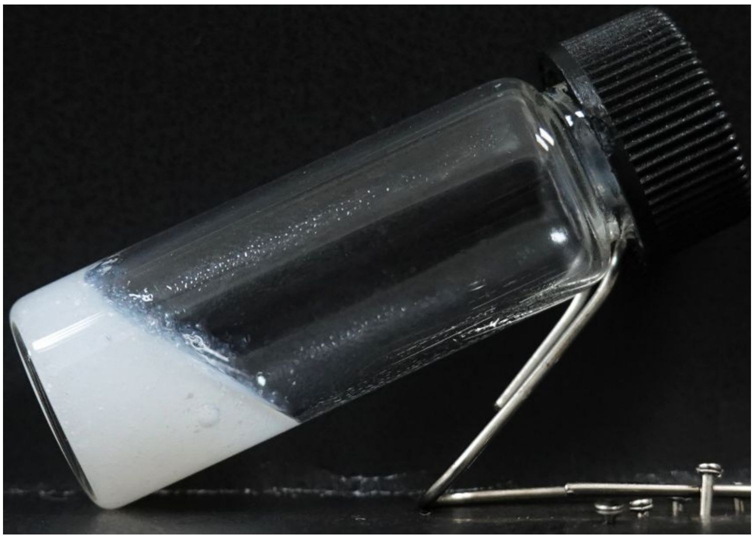

Fig. S23. Photograph of the PTX@HA (prepared with 5.0 mg·mL^{-1} PTX and 20.0 mg·mL^{-1} HA).

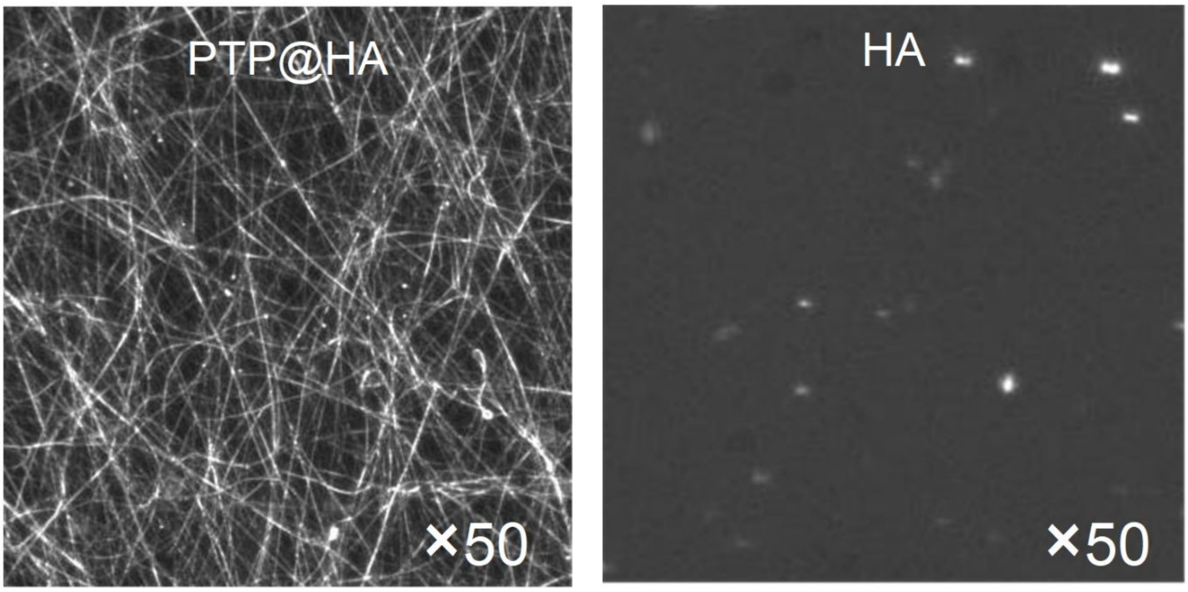

Fig. S24. Polarizing microscope images of PTP@HA (prepared with 5.0 mg·mL^{-1} PTP and 20.0 mg·mL^{-1} HA) and HA (20.0 mg·mL^{-1}).

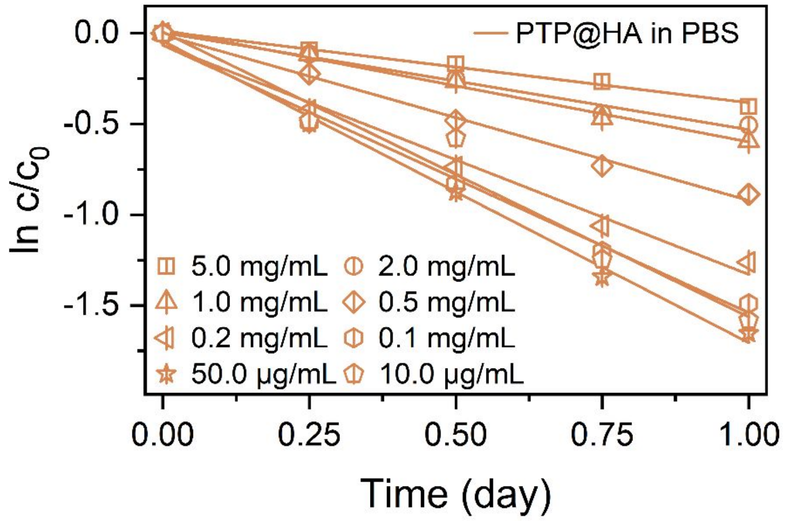

Equation	y = a + b*x							
Concentration	5.0 mg/mL	2.0 mg/mL	1.0 mg/mL	0.5 mg/mL	0.2 mg/mL	0.1 mg/mL	50.0 μg/mL	10.0 μg/mL
Intercept	0.011	0.005	0.020	-0.008	-0.065	-0.065	-0.042	0.008
Slope	-0.393	-0.536	-0.618	-0.913	-1.264	-1.475	-1.663	-1.571
R²	0.988	0.983	0.992	0.994	0.986	0.991	0.995	0.965

Fig. S25. Degradation kinetics of PTP@HA in PBS at various initial concentrations. The plot shows the natural logarithm of the concentration ratio ($\ln(c/c_0)$) versus time (days) for initial PTP@HA concentrations ranging from 10.0 μg·mL^{-1} to 5.0 mg·mL^{-1}. Solid lines represent linear regression fits to the data. The table below provides the equation parameters.

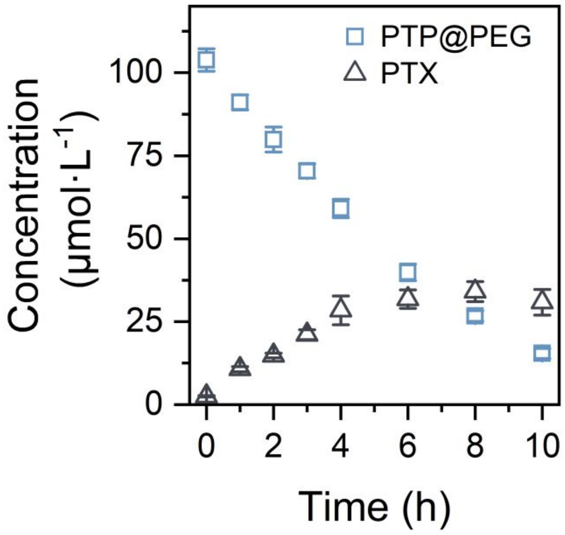

Fig. S26. *In vitro* degradation of PTP@PEG (100 μg·mL^{-1} PTP-equivalent) in plasma.

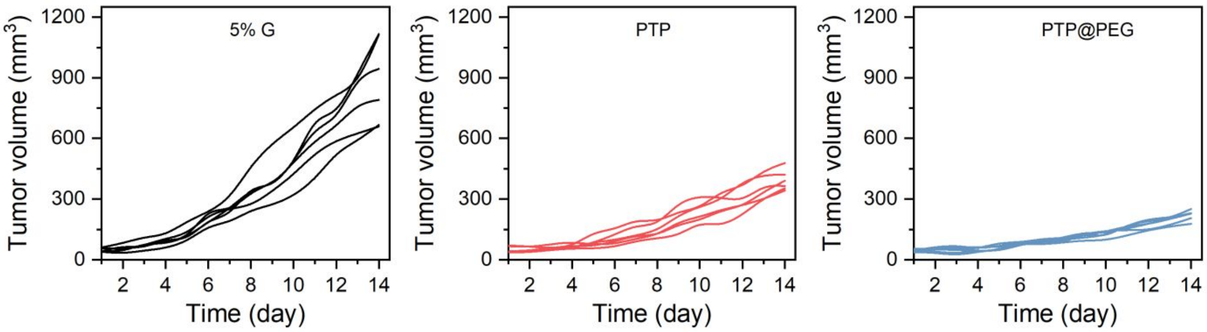

Fig. S27. Individual tumor growth curves in mice from different treatment groups. Panels show tumor volume (mm^3) trajectories over time (days) for individual mice treated with (left) 5% glucose (5% G, control), (middle) PTP, and (right) PTP@PEG. Each line represents data from a single animal (*n*=6 pre group).

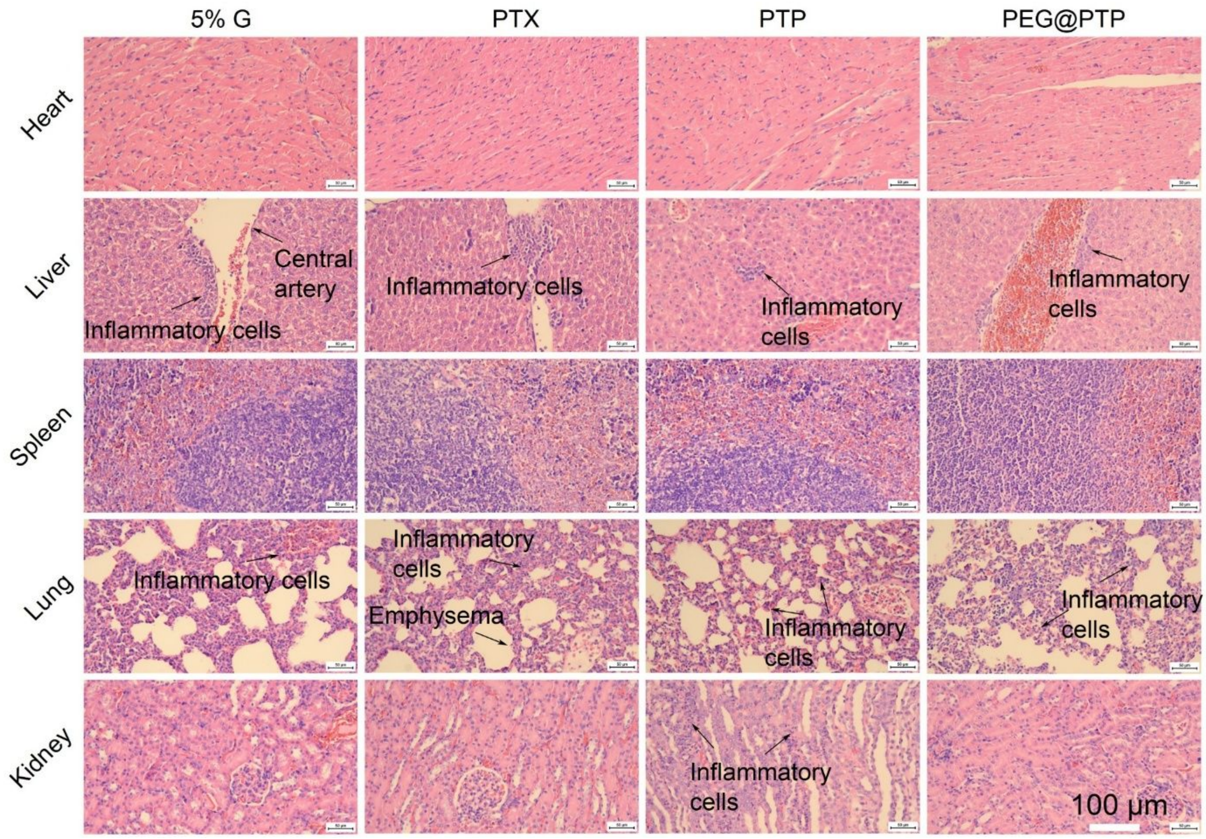

Fig. S28. Histological analysis of major organs after treatment. Representative HE-stained sections of heart, liver, spleen, lung, and kidney from mice treated with 5% glucose (5% G, control), PTX solution, PTP, or PTP@PEG are displayed.

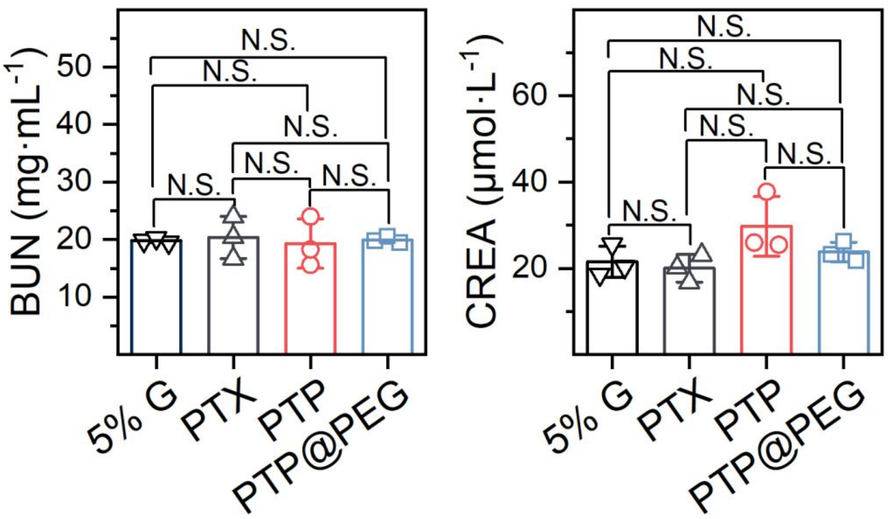

Fig. S29. The BUN and CREA of 5% G, PTX solution, PTP and PTP@PEG groups. (*N.S.*, not statistically significant).

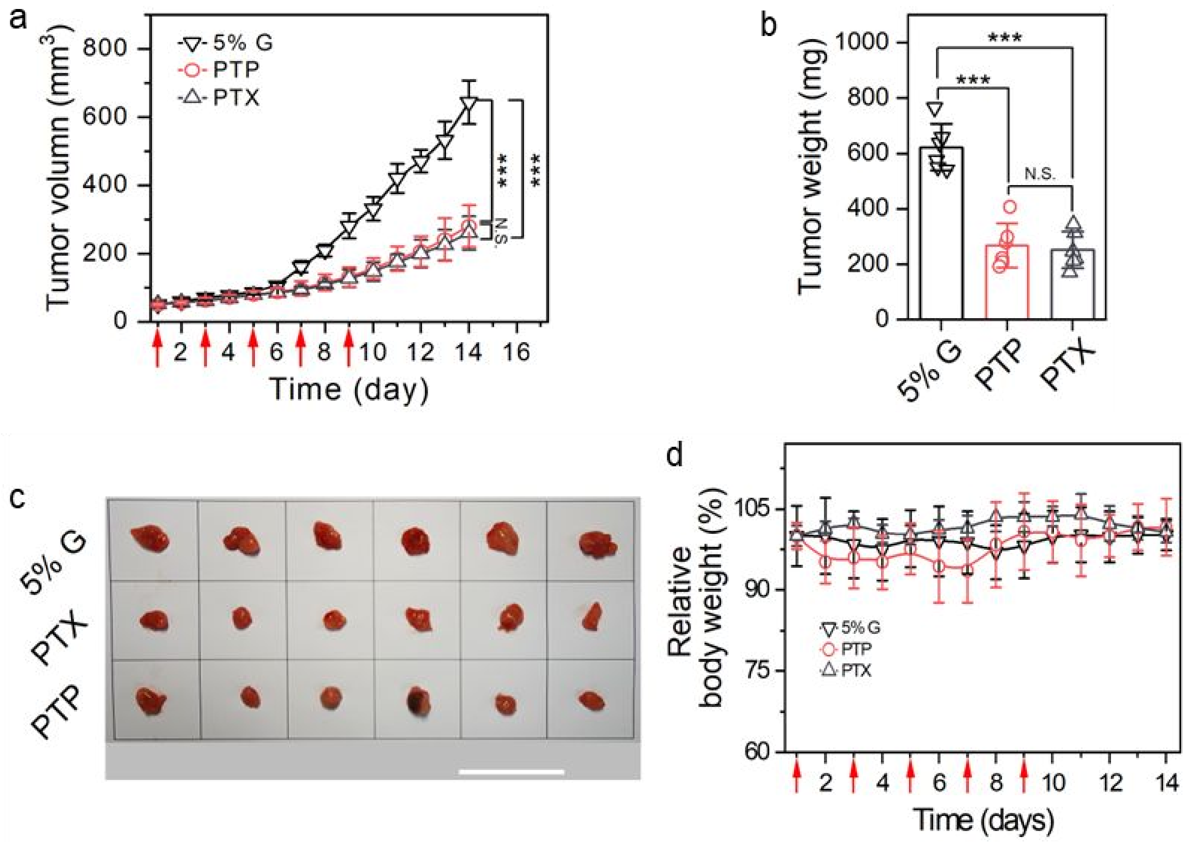

Fig. S30. Evaluation of anti-tumor efficacy and systemic toxicity for PTP versus PTX solution. Treatments were administered at 10.0 mg·kg^{-1} (PTX equivalent dose) according to the schedule indicated. (**a**) Average tumor growth curves (volume *vs* time). Red arrows indicate administration days. (**b**) Average tumor weight at the study endpoint. (**c**) Representative photographs of excised tumors at the study endpoint. (**d**) Relative body weight changes over the experimental period. *N.S.*, not statistically significant and $^{***}p < 0.001$.

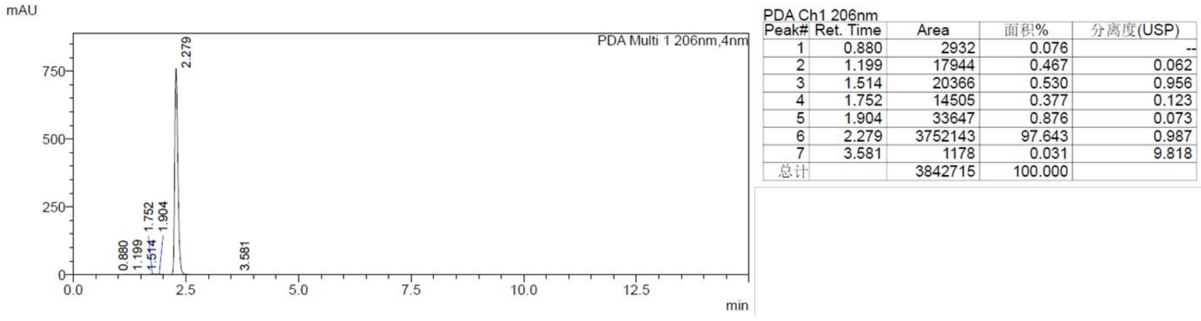

PDA Ch1 206nm

Peak#	Ret. Time	Area	面积%	分离度(USP)
1	0.880	2932	0.076	--
2	1.199	17944	0.467	0.062
3	1.514	20366	0.530	0.956
4	1.752	14505	0.377	0.123
5	1.904	33647	0.876	0.073
6	2.279	3752143	97.643	0.987
7	3.581	1178	0.031	9.818
总计		3842715	100.000	

Fig. S31. HPLC chromatogram of BPA, recorded at 206 nm. Retention times and peak areas are indicated in the inset table.
